# Experimental and Computational Approaches to Identify RNA–Protein Interactions

**DOI:** 10.3390/cells15171546

**Published:** 2026-08-27

**Authors:** Brigette Romero, Jadira Aurora Fuentes Bautista, Victoria Beringer, Maryia Hrynashka, Vijay Parashar, Mona Batish

**Affiliations:** Department of Medical and Molecular Sciences, University of Delaware, Newark, DE 19716, USA; bmromero@udel.edu (B.R.); jadira.fuentes@hotmail.com (J.A.F.B.); vbering@udel.edu (V.B.); marhry@udel.edu (M.H.); parashar@udel.edu (V.P.)

**Keywords:** RNA-binding proteins, RBP identification, RNA–protein interactions, experimental identification methods, computational prediction methods, crosslinking, proximity labeling, RNA-centric and protein-centric approaches

## Abstract

**Highlights:**

**What are the main findings?**

- Evaluates both experimental and computational methods for identifying RNA–protein interactions, detailing their pros and cons.
- Provides a practical framework to help researchers choose the best method based on cost, throughput, and sample needs.

**What are the implications of the main findings?**

- Combining computational predictions with experimental testing creates a highly accurate way to map complex RNA–protein networks.
- Better interactome mapping will accelerate clinical biomarker discovery and targeted therapies for various diseases.

**Abstract:**

RNA-binding proteins (RBPs) are essential regulators of RNA metabolism and gene expression, influencing processes such as splicing, stability, localization, and translation. Despite their critical roles in health and disease, including cancer, identifying RNA–protein interactions remains challenging due to technical limitations and biases of existing methods. Here we review and compare experimental techniques—including in vitro affinity purification, in vivo crosslinking, and proximity labeling—and computational prediction tools for RBP identification. We assess their strengths, limitations, and applicability across biological contexts, emphasizing the benefits of integrating experimental and computational strategies. Our analysis provides practical guidelines for selecting appropriate methodologies tailored to different cell types and research goals. These insights aim to facilitate more accurate mapping of RNA–protein interactomes, thereby advancing understanding of RBP functions and supporting the development of novel therapeutic interventions targeting RNA–protein complexes.

## 1. Introduction

RNA molecules, including protein-coding mRNAs and diverse non-coding species such as microRNAs (miRNAs), small interfering RNAs (siRNAs), small nuclear RNAs (snRNAs), long non-coding RNAs (lncRNAs), and circular RNAs (circRNAs), are dynamic regulators of cellular homeostasis. Rather than acting alone, RNA molecules function within complex, highly orchestrated ribonucleoprotein (RNP) complexes, where they are almost always bound by numerous RNA-binding proteins (RBPs) that play essential roles in protecting and regulating RNA. RBPs constitute approximately 5–10% of the human proteome, and approximately 4000 potential human RBPs have been identified through computational predictions and mass spectrometry approaches, although a majority of these remain functionally uncharacterized [1,2,3,4,5].

**Figure 1:**
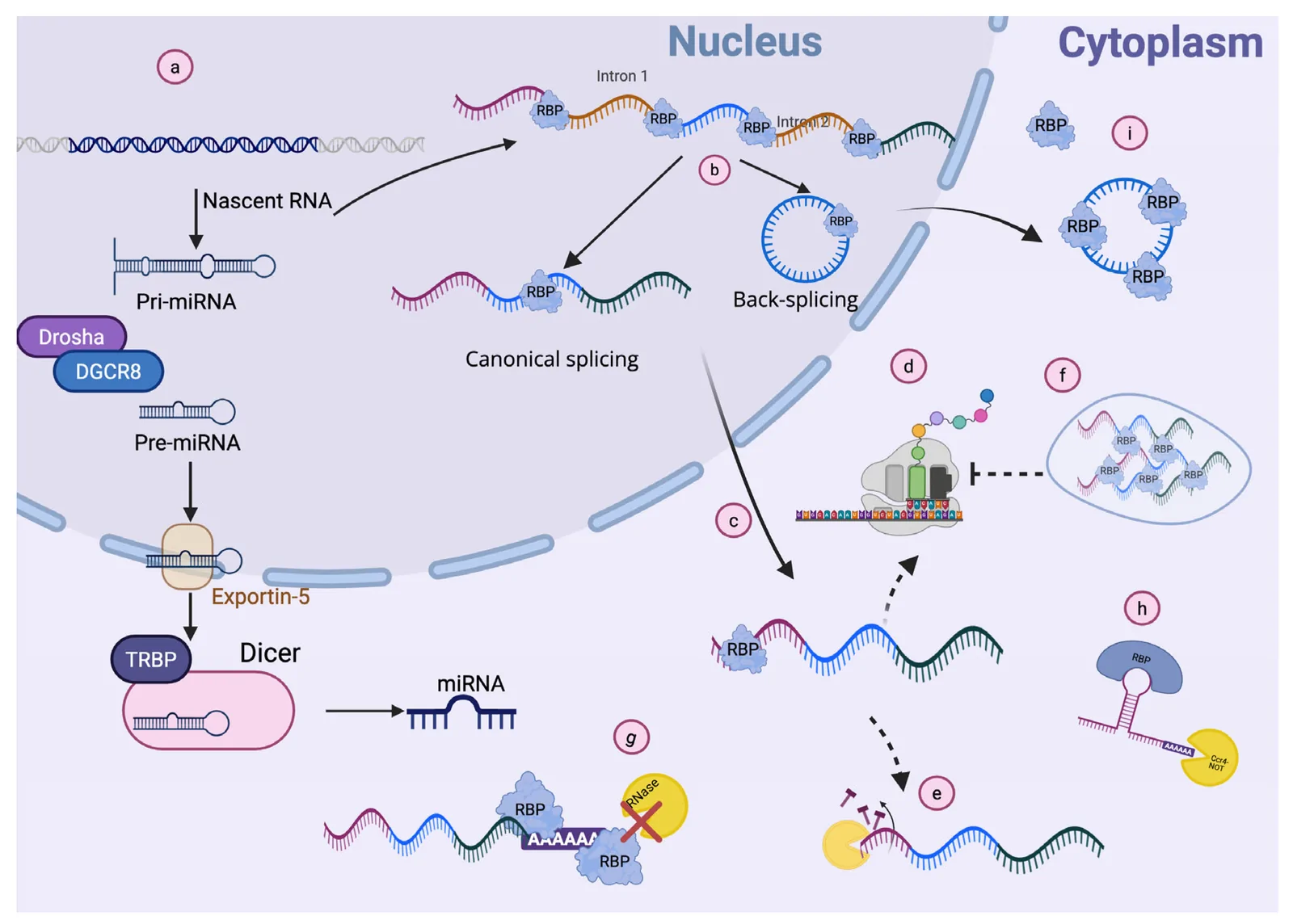
Potential functions of RBPs in RNA life cycle. RBPs modulate the RNA life cycle through diverse mechanisms, including (**a**) regulation of pre-miRNA biogenesis by proteins such as Drosha and Dicer [11]; (**b**) regulation of canonical splicing and back splicing to generate mature mRNAs and circRNAs, respectively, via exon inclusion or exclusion [12,13]; (**c**) mediation of mRNA localization by guiding transcripts to specific cellular destinations through the binding of RBPs to signaling sequences; (**d**) enhancement or repression of translation; and (**e**) RNA decay [14]. RBPs regulate translation by (**f**) acting as scaffolds for stress granule formation, thereby inducing translational stalling [15]. RBPs can promote mRNA stabilization by (**g**) binding to the poly(A) tail to shield it from RNases, or trigger degradation by (**h**) recruiting RNases to short hairpin sequences [12]. Separately, (**i**) circRNAs can serve as decoys that sequester RBPs in specific cellular locations [13]. The RBPs shown here depict both direct binding and complex-mediated mechanisms for simplicity. Created with BioRender.com (accessed on 21 February 2026).

RBPs regulate a wide range of cellular processes, including RNA processing, export, localization, translation, decay, splicing, polyadenylation, stability, and interactions with their partners [1,2,4,6,7,8,9,10]. They also play roles in the folding and assembly of ribosomal RNA–protein complexes and the processing of small nuclear RNAs [7]. Figure 1 illustrates an example of the mRNA life cycle to highlight how the RBPs dynamically regulate processing, transport, translation, and decay across distinct cellular compartments.

RBPs interact with both coding and non-coding RNAs to control RNA function [2,9,16,17,18]. Notably, RBPs have been reported to influence circRNA biogenesis and catabolism [16]. When circRNAs interact with RBPs, they can modulate the function of these proteins by acting as decoys or transporters; in some cases, circRNAs can also “sponge” RBPs, thereby modulating their activity [19,20].

Although much progress has been made in identifying RBPs, the precise mechanism by which they selectively bind their RNA targets remains only partially understood [2,21]. Despite the diverse structures and functions of RBPs, they typically contain at least one RNA-binding domain (RBD), a conserved protein domain that enables recognition of specific RNA sequences, motifs, secondary structures, or chemical modifications [1,2,4,22,23,24]. Many RBPs possess multiple RBDs or form complexes with other RBPs, allowing them to bind RNA in a multivalent and cooperative manner, which confers higher specificity and affinity compared to single RBDs; notably, bivalent binding is a common strategy among RBPs that enhances both affinity and sequence specificity [4].

Intrinsically disordered regions (IDRs), such as arginine-glycine-glycine (RGG) motifs and tyrosine-rich regions, are also prevalent in RBPs and contribute to RNA binding. Due to their lack of stable structure, structural studies have been focused primarily on the more ordered parts of RBPs, and many RBPs still lack well-characterized RBDs. In addition to an RBD, the most well-known RBPs also include K homology domains (KH), double-stranded RNA-binding domain (dsRBD), DEAD (Asp-Glu-Ala-Asp) box helicase domains, cold-shock domain (CSD), zinc fingers (ZnF), pumilio homology domain (PHD), and IDRs themselves [2,7,17,22,23]. Interactions between RBDs and RNA occur through diverse mechanisms, such as hydrogen bonds, van der Waals forces, salt bridges, aromatic stacking, and hydrophobic interactions [21,23].

RBPs have increasingly been recognized as key players in disease development, including genetic diseases, cancer, inflammatory diseases, metabolism-related diseases, and neurodegenerative disorders, and are promising targets for drug discovery, as shown in Supplementary Table S1 [2,6,7,23,25,26,27]. Genetic mutations in RBPs and abnormal expression have been detected in multiple human tumor types, where cancer cells exploit the post-transcriptional gene regulation of RBPs to modulate protein expression levels and sustain oncogenic proliferation [7,9,28]. Although the roles of splicing-regulatory RBPs in apoptosis, angiogenesis, proliferation, and metastasis are increasingly recognized, thousands of novel RBPs in cancer remain poorly understood. Moreover, RBPs can switch between oncogenic and tumor-suppressor roles depending on the specific context within cancer cells [21]. Therefore, new methodologies that enable characterization of roles of RBPs in cancer and identification of their binding sites are still needed to deepen our understanding of tumor biology and identify new therapeutic targets [3,25,28]. Developing RBP inhibitors remains challenging due to the conformational flexibility of RBPs and the lack of well-defined binding pockets [21].

In recent years, several databases of putative RBP binding sites and partners have been compiled to facilitate RBP identification and to help narrow down target lists prior to experimental validation. However, these databases include only a small number of experimentally validated RBPs and their respective targets [29], highlighting the need for continued experimental efforts.

In this review, we focus on RNA-centric methods developed over the last five years for RBP-RNA identification, while also including protein-centric methods and a brief description of foundational approaches for comparison and in clinical/low-input contexts. With the current rise in the use of RBPs as therapeutic targets, methods to discover and validate binding partners are crucial for developing novel treatments. Two often complementary approaches are used to identify RBPs, including RNA-centric and protein-centric methods. RNA-centric methods involve identifying the proteins bound to a specific RNA molecule (e.g., RNA-immunoprecipitation, crosslinking, and proximity labeling), while protein-centric methods identify the RNA targets of a specific RBP of interest, including methods such as CLIP, TRIBE, and STAMP, among others [30]. One of the most commonly used identification methods is CLIP (Crosslinking and Immunoprecipitation), which requires UV crosslinking of RNA–protein complexes [31] for identifying RNA-RBP interactions. It employs UV crosslinking prior to cell lysis to prevent adventitious interactions, such as those between cytoplasmic RBPs and nuclear RNAs in different biological contexts [1,6,10,25]. CLIP has the potential to result in high background due to nonspecific antibody–antigen interactions. The method is labor-intensive, requires significant financial resources and demands substantial starting material because of low crosslinking efficiency and the loss of RNA during immunoprecipitation [1,6,16,17,21,23]. Additionally, the RNA digestion step of the protocol complicates the identification of RNA isoforms and makes it challenging to determine the dynamics of ribonucleoprotein complexes (RNP complexes) [6,10,25]. To overcome many of these limitations, more than 20 CLIP variants have been developed over the years, with iCLIP, PAR-CLIP, eCLIP, and HITS-CLIP being among the most widely used [32]. Unlike traditional CLIP methods that use antibodies, variants such as iCLAP and uvCLAP utilize affinity-tagged RBPs. While this allows for the removal of co-purified RBPs, it can also lead to artifacts associated with overexpression [33]. Therefore, new methods are needed to reduce complexity, bias, and the number of experimental steps, as well as to identify high-confidence targets in endogenous biological contexts without starting material serving as a limiting factor [1,6,25,32].

Despite its widespread use, UV crosslinking has been reported to introduce biases into interaction profiles due to its intrinsic chemical properties [31]. Alternatively, antibody-based pull-downs of protein–RNA complexes, such as RIP-seq, are limited to a narrow set of interactions due to their lack of scalability for high-throughput analysis [31]. Other common methods, including poly-A pull-down, are unable to capture non-polyadenylated species such as lncRNAs, miRNAs, and histone mRNAs [31]. Proximity-based labeling enzyme techniques can also yield false positives by capturing proteins that are merely in the vicinity of the RNA of interest rather than directly interacting with it [34]. Both proximity biotinylation and UV crosslinking methods are prone to false positives and false negatives, highlighting the need for more stringent identification approaches [35]. Furthermore, in situ observations of RBP interactions remain challenging because many methods require the use of cell lysate [36]. Many of these approaches are also costly and time-consuming, which hinders the discovery and validation of RNA-RBP interactions [37]. Collectively, these limitations underscore the urgent need for new methods to identify RNA–protein binding partners, as they create a bottleneck for advancing novel treatments.

### The Methodological Challenge in Mapping RBP Dynamics

Despite the critical importance of RBP-RNA networks, comprehensively mapping these interactions remains a major experimental and computational hurdle. Classical biochemical approaches, such as RNA Immunoprecipitation (RIP) and traditional Crosslinking and Immunoprecipitation (CLIP) and its variants (e.g., iCLIP, eCLIP), revolutionized the field by providing genome-wide maps of RBP binding sites. However, these foundational techniques present well-known trade-offs, including labor-intensive protocols, large sample inputs, and non-specific interactions [38,39]. Most of the traditional methods lack a comprehensive approach and are limited to providing static snapshots; methods capable of capturing the dynamic interactions of RBPs within tumors are still needed.

Recent developments have shown that novel sequencing methodologies can be used to directly map in vivo RBP-RNA interactions using crosslinking, purification, and ligation techniques. These interactions are regulated by cooperative and competitive binding events that govern gene expression. These approaches help bridge the gap between biochemical accuracy and physiological relevance [40].

The use of a broad range of testing methods and advanced tools will help better understand the complexity of RBP interactions, as these approaches can capture changes in binding partners in response to cellular stress, development, or cancer-related signals [41].

Emerging modeling techniques and machine-learning-based binding predictions are now being used to predict RBP preferences and binding rules, such as those of Pumilio-family proteins. However, the accuracy of such models depends on the quality and diversity of the available experimental data; hence, continued methodology development is greatly needed [42].

However, methodological advances also open clinical perspectives. For example, the development of peptide nucleic acids that mimic the structure of RNAs and inhibit RBPs presents new tools for probing and modulating RBP-RNA interactions, thereby offering potential therapeutic applications [43].

In the long run, integrating approaches such as high-resolution sequencing, structural modeling, and live-cell imaging may facilitate the transition from static catalogs to dynamic networks of RBP interactions and could provide insight into how RBP complexes are reorganized in real time during tumor development or during resistance to therapy. Further development of these tools is not only a technical advance but may also be important for understanding how RNA–protein interactions support cancer cell identity and for translating this knowledge into clinical benefit. Here we provide a critical, comparative synthesis of current experimental and computational strategies. By evaluating methods across standardized performance metrics, such as input requirements, resolutions, throughput, and error rates and integrating them with clinical contexts, we aim to offer a practical framework to guide experimental design.

## 2. Comparative Analysis of Methodological Approaches

RNA-binding protein (RBP) identification may be achieved through different methodological categories: in vitro, in vivo, in situ, and in silico. In vitro, or “test tube” methods, such as RNA pull-down, involve using purified components to study the relationships between proteins and RNAs. These methods are typically able to provide high-resolution and quantitative data. The experiments are performed under highly controlled conditions and can provide clear results, but a drawback is the lack of physiological relevance.

On the other hand, in vivo methods are used to capture interactions as they occur naturally within a living cell, rather than in a test tube. They can provide a transcriptome-wide map of RBP targets. Using in vivo methods offers higher biological relevance, but they are often associated with more complex protocols, require specific antibodies, and yield data that may be compromised by background noise. 

**Figure 2:**
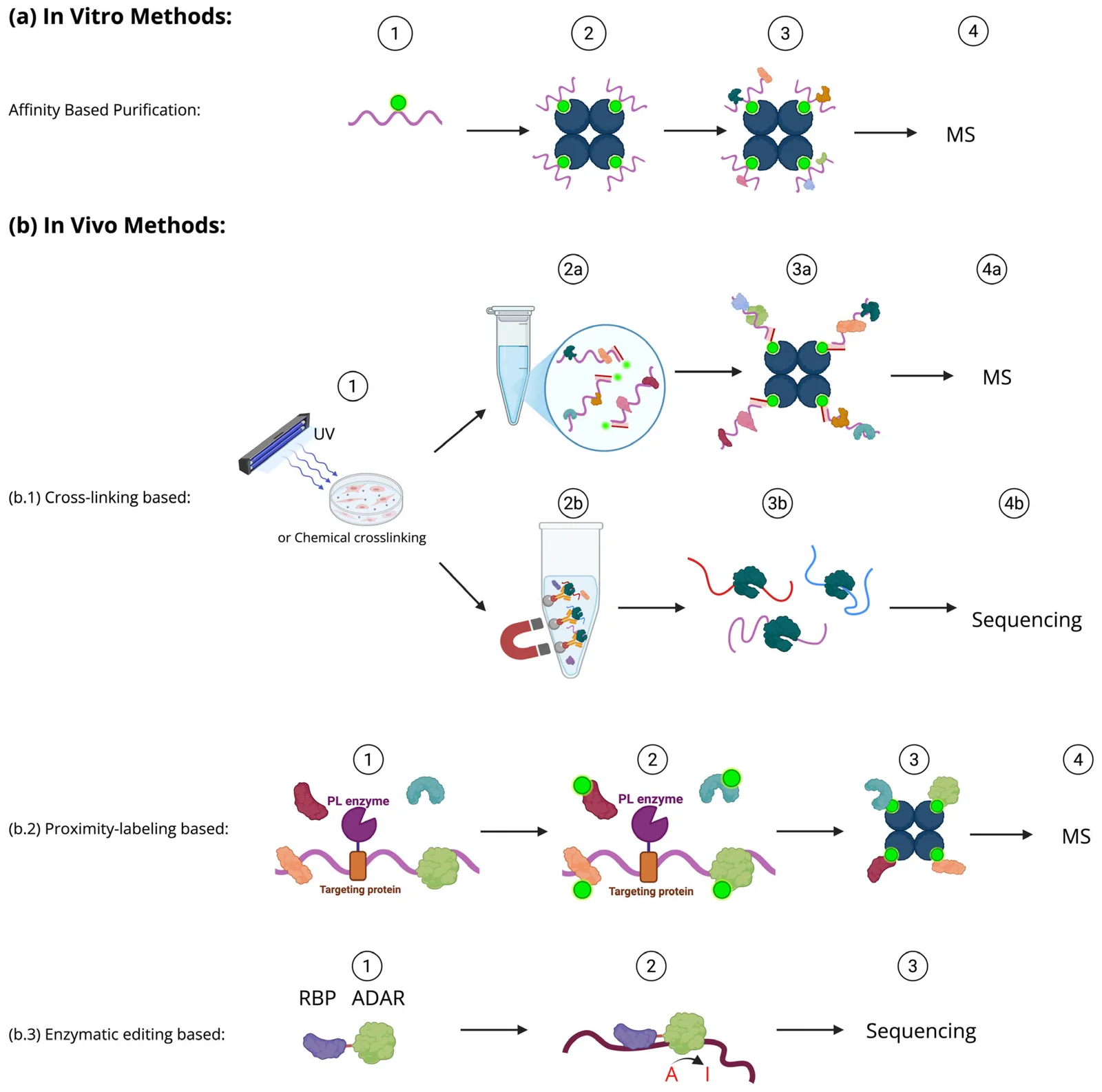
General overview of in vitro and in vivo methods. (**a**) In vitro methods for identifying RNA–protein interactions often rely on RNA pull-down assays. ((**a**) 1–4) In vitro-transcribed, biotinylated RNA is coupled to streptavidin beads and incubated with cell lysates. Beads are washed and boiled to elute-RNA protein complexes for downstream MS analysis. (**b**) In vivo methods, on the other hand, rely on (**b.1**) crosslinking-based methods, (**b.2**) proximity-labeling-based techniques, and (**b.3**) enzymatic editing methods. In crosslinking-based approaches, focusing on RNA-centric methods ((**b.1**) 1–4a), RNA-RBPs complexes are crosslinked either with UV light or with formaldehyde, captured using biotinylated antisense probes, and purified with streptavidin beads for analysis via mass spectrometry. Conversely, in protein-centric methods based on crosslinking ((**b.1**) 1–4b), the RBP-RNA complexes are crosslinked, a selected RBP is immunoprecipitated, and the bound RNA is identified via sequencing. Regarding proximity-labeling-based (PL) techniques ((**b.2**) 1–4), a fusion protein targets specific RNA. Upon adding a biotin substrate (or an equivalent), the PL enzyme labels nearby molecules, which are then captured by streptavidin beads and eluted for analysis. Finally, enzymatic editing methods ((**b.3**) 1–3) such as TRIBE function by fusing the RBP of interest to the enzyme ADAR (Adenosine Deaminase Acting on RNA), which deaminates nearby adenosines, and these altered bases are later detected using [44]. Created with BioRender.com (accessed on 21 February 2026).

As illustrated in Figure 2, in vitro techniques rely on RNA-pull-down assays to isolate RBP complexes from cell lysates, whereas in vivo methods are much more diverse, encompassing crosslinking methods to directly capture interacting molecules, proximity-labeling strategies to tag adjacent proteins in their native environment, and enzymatic editing techniques that map interactions by actively modifying nearby RNA bases.

In situ methods represent a “middle ground” between in vivo and in vitro approaches. These techniques study the RNA-RBP complexes through fixed cell or tissue samples. The natural context of the molecules is largely preserved, while the complications and limitations associated with live-cell and in vitro experimentations are minimized. 

In silico methods are computational approaches that rely on machine learning and bioinformatics to predict RBP relationships. Most of the data used in these methods are generated via experimental approaches, and the predictions from this data can be confirmed through the same methods. The biggest advantage of these methods is their high throughput and cost-effectiveness. This can allow scientists to predict RBP-RNA interactions by screening entire genomes. On the other hand, a major limitation is that the results of these methods must still be experimentally validated to be considered accurate and reliable. 

### 2.1. In Vitro Methods to Identify RBPs

The identification of RBPs in vitro is often achieved through affinity purification methods, most often through the RNA pull-down assay (Table 1). This method utilizes a specific RNA of interest to “pull down” the interacting proteins from a cell lysate. The main components of this technique include attaching a high-affinity tag, most commonly biotin, to the RNA of interest, followed by purifying the RNA-RBP complexes with streptavidin-coated beads. This method is effective due to the high affinity between biotin and streptavidin. These steps are then followed by washing, elution, and identification of the proteins, typically via mass spectrometry (MS). 

**Figure 3:**
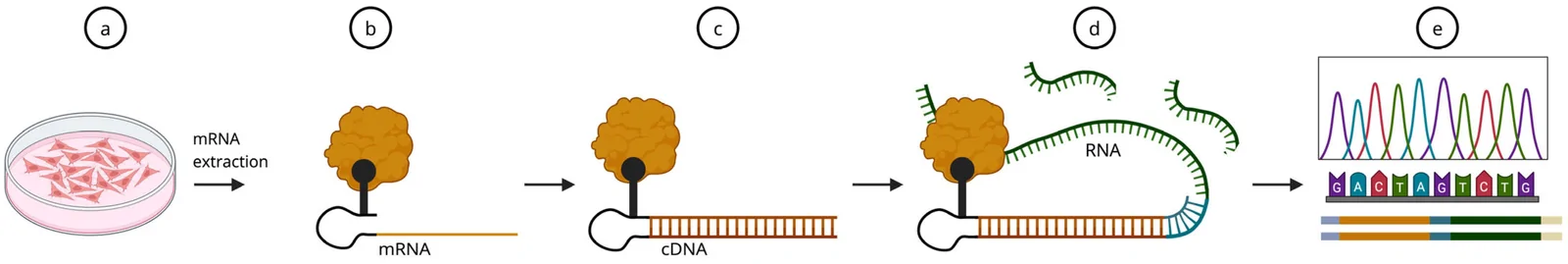
Experimental workflow of PRIM-seq method. (**a**) Total RNA is isolated from cultured cells, and (**b**) a puromycin-conjugated linker is attached to the 3’ ends to enable in vitro translation of covalently linked mRNA–protein fusions. (**c**) The mRNA barcodes are reverse-transcribed into cDNA, yielding a library of cDNA-labeled proteins. (**d**) This library is incubated with a target RNA library to allow for RNA–protein interactions, followed by ligation to generate chimeric molecules. (**e**) The resulting chimeric products are sequenced, enabling the identification of RNA-RBP interactions [45]. Created in BioRender.com (accessed on 21 February 2026).

In vitro methods include miTRAP and PRIM-seq. Figure 3 presents the general workflow for PRIM-seq, a method for identifying RBPs and their interacting RNAs de novo by performing two experimental modules: SMART-display and REILIS. The first module, SMART-display, consists of extracting mRNA from input cells, adding a linker to the 3’ of each mRNA, and translating the mRNA to create proteins covalently linked with the mRNA. The second module is REILIS, which stands for reverse transcription, incubation, ligation, and sequencing. The mRNA end of the labeled proteins is converted to cDNA; this complex is then incubated with an RNA library to allow RNA–protein interactions. The RNA and cDNA labels are ligated into a chimeric sequence, forming an RNA-linker-cDNA complex, which is then subjected to paired-end sequencing. The cDNA part reflects the protein, and the RNA reflects the interacting RNA. Finally, PRIM-seq resolves the RNA–protein interactions from the chimeric sequences using bioinformatics.

Although PRIM-seq offers a robust system for mapping reconstituted bindings rather than endogenous cellular occupancy, there are some limitations, including random priming, which can lead to truncated proteins, and nucleic acid conjugation, which can affect protein folding and RNA binding. The in vitro conditions cannot fully replicate the complexities of the intracellular milieu, and they may yield false positives and false negatives. Consequently, orthogonal approaches, such as targeted mutagenesis, established RNA–protein interaction assays, and cellular assays that capture native binding, should be used in conjunction with PRIM-seq to validate and confirm the biological significance of the findings [45,46].

The miTRAP (miRNA trapping by RNA in vitro affinity purification) method is another example of an affinity-based approach for RBP identification. This method acts effectively by employing an MS2 tag system. The MS2 tags are attached to the RNA of interest and mixed with amylose resin beads and a fusion protein. The fusion protein consists of the MS2-binding protein and maltose-binding protein. The MS2 binding protein can attach to the RNA of interest and capture its interactions, while the maltose-binding protein has a strong affinity to the amylose resin beads. This interaction immobilizes the RNA, and any unspecific binding can be washed away, leaving the purified RBP-RNA interactions. The RBPs can be analyzed through mass spectrometry or Western blotting [47].

**Table 1:** In vitro methods.

Method	Description	Advantages	Limitations	Cost *	Time	Throughput **	Citation
miTRAP	Uses an MS2 tag system and amylose resin beads for affinity-based purification to identify miRNAs and RBPs interacting with a target RNA.	Utilizes commercially available components; employs a simplified cloning strategy.	Lacks an endogenous cellular environment; limited primarily to miRNAs; potential for unspecific binding.	$$	5–7 days	Low	[47]
PRIM-seq	Employs SMART-display to barcode proteins with their corresponding mRNA, alongside REILIS to create dsDNA sequences containing the cDNA of both the mRNA and the interacting RNA.	Identifies RBPs and associated RNAs at the genome scale; enables de novo identification of interactions; requires no specific reagents to target specific proteins or RNAs.	Lacks ability to replicate native cellular conditions; misses post-translational modifications and protein function; lacks suitability for detecting direct interactions; risks false positives and false negatives.	$	3–5 days	High	[45]

Footnote: * Cost scale: Detailed as low ($), and moderate ($$), and based on the requirement for specialized reagents, such as individually biotinylated probes versus universal oligos. ** Throughput and time: Estimated turnaround times include cell preparation, crosslinking, purification, and mass spectrometry preparation phases. All abbreviations and their definitions and expanded forms are listed in Supplementary Table S2.

### 2.2. In Vivo Methods to Identify RBPs

Chemical/photoreactive-mediated crosslinking methods have been the gold standard for identification of RBPs in vivo. However, there has been a notable surge in the development of methods involving fusion proteins that have been developed to specifically target a molecule of interest. These fusion proteins involve a proximity-labeling enzyme to attach tags to the interactome, and a protein targeting the molecule of interest. 

#### 2.2.1. Crosslinking-Based Methods to Identify RBPs

Crosslinking methods can use formaldehyde/glutaraldehyde or UV light to covalently bind molecules to each other, like RNA and proteins (Table 2). To enhance the capture of RNA–protein interactions, both crosslinkers require high input cell numbers, from 10^8^ to 10^9^ cells. Historically, RNA-centric MS pull-down methods for low-abundance targets required extremely high inputs, which typically had to be scaled based on RNA abundance and the chosen readout. To only extract the RNA–protein crosslinked complexes, RNA is purified under denaturing conditions to remove noncovalent interactions, and crosslinked proteins are extracted for subsequent identification [44].

UV light, compared to formaldehyde, is a zero-distance cross linker for direct contacts, while aldehydes readily generate protein–protein bridges that could blur direct contacts. Nonetheless, crosslinking with UV light is generally less efficient. Also, UV crosslinking has a bias towards uridine, and double-stranded RNA (dsRNA) is poorly crosslinked. The efficiency of UV crosslinking varies by amino acid and may also depend on the structure and surface area of RNA–protein complexes [44].

Formaldehyde is a small aldehyde that acts as a bifunctional crosslinker, permeating cells and covalently linking macromolecules within 2 Å in a reversible manner. Formaldehyde crosslinking has a bias toward nucleophilic lysine residues. This compound also forms covalent bonds between proteins in addition to RNA–protein complexes, making it difficult to distinguish between proteins that interact directly with RNA and those that form complexes with bound proteins [44].

**Figure 4:**
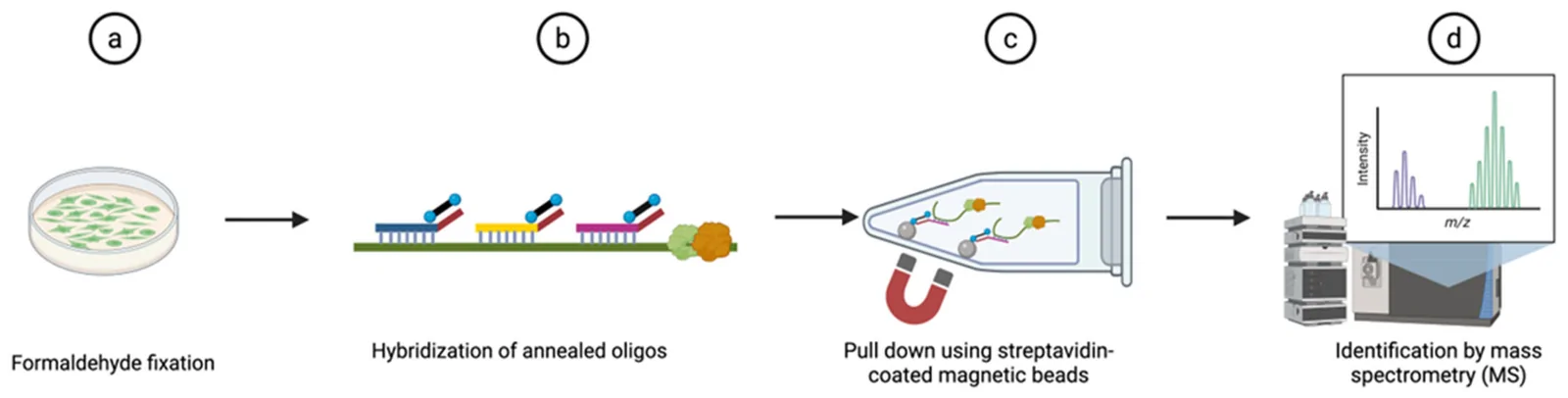
Experimental protocol for MORPH-MS. (**a**) Cells used for each pull-down, including the odd probe, even probe, and RNase A-treated groups, are crosslinked in 3% paraformaldehyde in PBS and then subjected to chromatin shearing. (**b**) The complementary probes, divided into odd and even groups, are annealed with a universal oligo, and subsequently, the annealed probes are added to the samples to hybridize. (**c**) The pull-down is performed using streptavidin-coated magnetic beads. (**d**) After the beads are washed, the proteins are eluted with benzonase and identified by mass spectrometry [51]. Created in BioRender.com (accessed on 21 February 2026).

Among the methods described above, MORPH-MS (Multiple Oligo-assisted RNA Pull-down via Hybridization followed by Mass Spectrometry) and LEAP-RBP (Liquid-Emulsion-Assisted Purification of RNA-Bound Protein) are examples of newer approaches that are cost-effective and simple in vivo approaches to identify RBPs. MORPH-MS is a crosslinking method used to purify and identify proteins associated with a target RNA within the cell (Figure 4). The method relies on an array of 20–25 antisense oligos that are collectively complementary to the entire length of the target RNA. These tiling oligos are designed to provide one oligo per 350–450 nucleotides of RNA length, maintaining a GC content of approximately 48–52%. Their specificity is manually screened using NCBI blasts to select probes with an E-value below 0.0002. Additionally, all oligos share a common sequence at their 5′ end. This common binding sequence allows all antisense oligos to hybridize to a single biotinylated universal oligo. Experimental controls for this method include dividing the probes into separate odd and even groups, incorporating a RNase A control, and utilizing non-target oligos against LacZ. During the procedure, these probes are mixed with the cell lysate, where they bind to the target RNA, and capture the native protein interactions. The biotinylated universal oligo facilitates the purification of these complexes using streptavidin-coated magnetic beads followed by stringent washing steps. Finally, the elution step is performed with benzonase, and the proteins are identified by mass spectrometry. The use of only one biotinylated probe allows for a significant reduction in the price of the procedure [51].

The LEAP-RBP method offers another crosslinking based approach to capture the proteins that interact with RNA inside of living cells. Following the crosslinking, the cells are lysed in a guanidinium-based buffer. The lysate is then used to perform an acidic phenol–chloroform extraction, separating the material into distinct layers. The interphase layer, where the RNA–protein complexes are found, is collected and enriched by a lithium-based precipitation step. The purpose of this step is to selectively pull down the crosslinked RNA–protein products and eliminate any free proteins and other potential contaminants. The product is then treated with DNase to remove DNA, leaving only RNA-bound products. To complete the protocol, the proteins are analyzed by mass-spectrometry to be able to be identified. This method is suitable for mapping the entire RNA–protein proteome, but a key limitation is its inability to focus on a single target RNA [50].

#### 2.2.2. In Vivo Proximity-Labeling Methods to Identify RBPs

**Figure 5:**
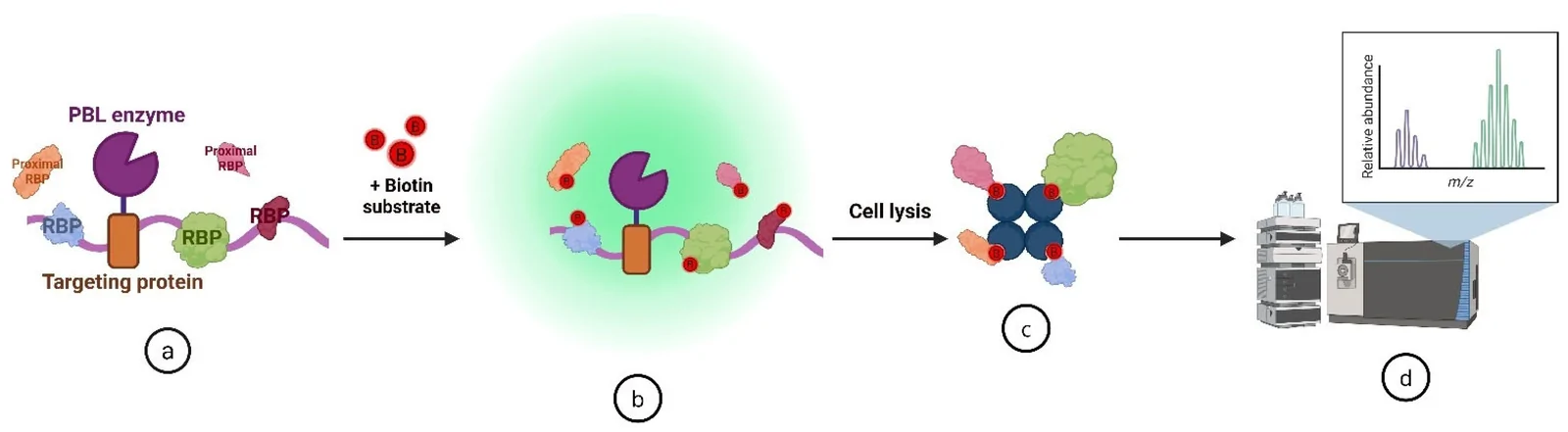
General workflow of proximity-based labeling. (**a**) The fusion protein targets the RNA of interest. (**b**) After a biotin substrate (or any substrate specific to the PL enzyme) is added, the PL enzyme biotinylates the surrounding molecules (including RBPs) within its labeling radius. (**c**) Subsequent to cell lysis, the lysate is incubated with streptavidin beads to enrich for biotinylated molecules. (**d**) Following incubation, the enriched molecules are analyzed by MS (e.g., LC-MS/MS) to elucidate their identity. Created with BioRender.com (accessed on 21 February 2026).

Proximity labeling (PL) techniques were introduced for the first time in 2012 as an alternative in vivo method to map molecular interactomes (Figure 5 and Table 3). Since then, various PL enzymes and methods have been developed to elucidate protein–protein, DNA–protein, and RNA–protein interactions. The principle behind this set of techniques is similar. A fusion protein that includes the PL enzyme and a protein that can be guided to the RNA of interest is generated. Once the fusion protein has encountered the target RNA, the cell is supplied with a substrate specific to the PL enzyme, which is then covalently attached to the nearby interactome. These molecules are then isolated by affinity-based purification and analyzed by downstream methods depending on what is being studied [55].

There are two general categories of PL enzymes: peroxidases and biotin ligases. In the presence of hydrogen peroxide (H_2_O_2_), peroxidases convert a great variety of substrates into radicals. A widely used peroxidase is APEX2, a plant ascorbate peroxidase variant of APEX engineered to exhibit improved thermal stability and enhanced biotinylating activity [56]. APEX2 forms free radicals of biotin-tyramide (biotin-phenol; BP) in the presence of biotin-phenol and H_2_O_2_. These free radicals react with tyrosine residues on the surface of proteins within a labeling radius of 20 nm, forming covalent adducts. The biotinylated molecules can then be isolated using streptavidin beads and analyzed by downstream methods like mass spectrometry [57].

On the other hand, biotin ligases allow the biotinylation of surrounding molecules. These molecules can then be isolated through biotin affinity purification and identified through various downstream methods; for proteins, this would involve mass spectrometry. There is a wide array of biotin ligases utilized for RBP elucidation, such as BirA, BASU, BioID2, among others. Each enzyme has its own specifications and labeling radius. BASU, for example, is a 29 kDa enzyme designed from *Bacillus subtilis* with a 10 nm labeling radius. It allows for the study of RNA–protein interactions in vivo within 1 min. PL time is crucial to capture transiently interacting proteins, which is why enzymes like BioID2 (16–18 h) can lead to false-positive or false-negative results. As described before, the biotinylated interactome can then be isolated by and subsequently analyzed based on the needs of the study [56].

A PL enzyme that does not fit into these categories is PafA, utilized in CRUIS (presented in Table 3), which attaches PupE to the lysines of surrounding proteins. This pupylation-based interaction tagging (PUP-IT) enriches transient and weakly interacting proteins, detected by mass spectrometry. While the PafA ligase ensures high specificity, Pup is relatively big, which could prevent transmembrane diffusion thereby not allowing for the detection of interactions within organelles [56].

Some ways in which the PL enzymes can be targeted to an RNA of interest is through complexes such as MCP/MS2 RNA-stem-loop or CRISPR-Cas13/guide RNA (gRNA). MS2 RNA-stem-loops can be added to the RNA of interest so that the fusion protein containing the PL enzyme/MS2 protein (MCP-MS2 coat protein) recognizes the stem-loop(s) and sets the stage for PL [58]. CRISPR-Cas13/gRNA takes advantage of the endonuclease-deficient Cas13 (dCas13) and its ability to be directed to target RNA with a sequence-specific gRNA [59]. As mentioned beforehand, the fusion protein would now be targeted to the RNA of interest and can start the PL process.

**Table 3:** In vivo methods—proximity-labeling based.

Method	Description	Advantages	Limitations	Cost *	Time	Throughput **	Citation
Proximity-CLIP	Labels RNA (with 4SU) for enhanced crosslinking efficiency, utilizes a localization element (LE)-APEX2 fusion protein to label the RNA interactome; uses UV light to crosslinks protein–RNA complexes.	Can profile proteome and transcriptome, enables compartment-specific analysis, can capture short-lived RNA, offers high specificity, can identify precise binding sites.	potential for false positives; risks cellular toxicity from hydrogen peroxide	$$$	3–5 days	Moderate	[60]
RPL	Uses dCas13b targeted by sequence-specific gRNA; fuses to engineered ascorbate peroxidase APEX2 to biotinylate electron-rich amino acids.	Requires no crosslinking, sonication, or RNA engineering; works efficiently with low number of cells.	Risks oxidative damage, generates large complex size that may lead to reduced specificity, comparatively low efficacy.	$$	2–3 days	Moderate	[59]
CARPID	Employs dCasRx directed by sequence-specific gRNA; fused to engineered biotin ligase (BASU) to biotinylate proximal proteins that are then pulled down using streptavidin beads.	Eliminates the need for crosslinking, pre-labeling target RNA, MS2 insertion, or antisense probe design; preserves the physiology of transfected cells; detects weak or transient interactions; achieves potentially broad RNA coverage using multiple gRNAs.	May identify merely proximate proteins (false positives), can sterically hinder normal RBP binding or alter RNA localization, may create artifacts, long labeling time precludes capturing short-lived interactions.	$$	3–5 days	Moderate	[61]
CBRPP	Uses dCas13 and a custom crRNA to deliver a PBL enzyme (e.g., dPspCas13b-BioID2-NES) to target RNA, which then biotinylates surrounding proteins; incorporates BioID2 (a promiscuous biotin ligase) and an NES (Nuclear Export Signal) for cytoplasmic export of the fusion protein.	No crosslinking required, no need to pre-label target RNA, MS2 insertion, or designing of antisense probes; maintains natural structure of target RNA and avoids RNA degradation; captures weak and transient RNA–protein interactions.	May generate false positives from mere proximity, fusion protein may interfere with the binding of endogenous RBPs, long labeling time precludes capturing of short-lived RNA–protein interactions.	$$	3–5 days	Moderate	[62]
CRUIS	Uses dLwaCas13a to target specific RNA sequences; employs the proximity-labeling enzyme PafA to label surrounding RBPs by ligating PupE to lysine.	No need for crosslinking, no manipulation of RNA and reduced RNA degradation, captures weak or transient interactions.	May generate false positives, risks fusion proteins interfering with endogenous RBP binding.	$$	2–3 days	Moderate-high	[63]
MCP-APEX2	Conjugates the bacteriophage MS2 RNA stem loop; utilizes an MS2 coat protein-fused APEX2 (MCP-APEX2) to bind the stem loop while APEX2 promiscuously labels proximal proteins.	No crosslinking, short labeling time to enable analysis of dynamic RNA interactomes and reduce the toxicity of hydrogen peroxide addition to cells, more sensitive than dCas13-APEX2, detects weak or transient interactions.	Requires RNA modification, steric bulk of MS2 tags could block certain interactions, may result in false positives.	$$	1–2 days	High	[64]
dCas13-APEX2	Employs a dCas13d-dsRBD-APEX2 fusion protein where dCas13d (with gRNA) targets the RNA; dsRBD stabilizes the gRNA-target RNA duplex; APEX2 proximity-labels the RNA interactome.	Uses short labeling time to analyze dynamic RNA interactomes to avoid toxicity of H2O2; can detect weak or transient interactions. No crosslinking and tagging of target RNA is not required, provides higher protein specificity than MCP-APEX2.	May result in false positives due to dCas13d-dsRBD fusion blocking certain interactions.	$$	1–2 days	High	[64]
BioRBP	Fuses BirA (a biotin ligase) to the phage PP7 coat protein (PP7cp); targets and binds PP7 RNA stem-loop motifs.	Requires no crosslinking; detects weak or transient interactions; performs well for low-expression or unstable RNAs.	May generate false positives and overexpression artifacts; requires genetic modification; risks interfering with native interactions.	$$	2–3 days	High	[65]

Footnote: * Cost scale: moderate ($$), and high ($$$) based on the requirement for specialized reagents, such as beads, fusion protein constructs, etc. ** Throughput and time: Estimated turnaround times include cell preparation, crosslinking, purification, and mass spectrometry preparation phases. All abbreviations and their definitions and expanded forms are listed in Supplementary Table S2.

### 2.3. Quality Control in RNA-Centric Mapping

For RNA-centric probe pull-downs, the negative controls must involve using beads-only or no probe control to verify that proteins are not binding non-specifically to the coated beads or matrix [66]. Utilizing a scrambled non-targeting probe is required to ensure that the identified RBP is sequence or structure specific [44,66,67]. Furthermore, the use of different sets of probes against different regions of the targeted RNA helps evaluate the specificity and validate binding, while the pull-down targets themselves should be confirmed by using unrelated or endogenous RNAs as controls [66,67]. Incorporating an RNase step that digests all unprotected RNA and yields the RNA bound to the RBP serves as an additional experimental control [66,68].

For proximity labeling methods, an experiment with an empty gRNA-expressing vector can help to define the baseline of background biotinylation [61]. Other essential controls to evaluate include using a scrambled non-targeting guide RNA, an inactive enzyme, or adding no substrate for the enzyme to function [69,70]. Additionally, the labeling enzyme can be targeted to a different organelle or protein complex in addition to the primary target to verify localization specificity [71]. The checklist requires verifying other experimental controls, including non-crosslinked samples, non-immunoprecipitated samples, and lysates from RBP knockout samples [32]. Lastly, to properly validate the overall experimental setup and downstream proteomic analysis, RNA with known RBP partners must be included as positive controls, alongside validation by Western blot and qRT-PCR [44,66].

### 2.4. Other Experimental Methods to Identify RBPs

While often overlooked, there have been some advances in the development of in situ and ex vivo methods for identifying RBPs. These techniques are very diverse, each of them following its own principle. This creates a wide range of alternatives for identifying and cross-validating RBPs interacting with the target RNA, as shown in Table 4. For example, hybridization-proximity labeling (HyPro) relies on the hybridization of digoxigenin-labeled antisense probes to the RNA of interest. Then the HyPro enzyme, which is an APEX2/DIG10.3 fusion protein, binds to the digoxigenin probes. Any unbound enzyme is washed off, and the interactome of the RNA of interest is biotinylated in situ. After crosslink reversal, the labeled interacting molecules are captured on streptavidin beads and analyzed by MS. While similar to the in vivo PBL methods, HyPro-MS utilizes chemically fixed cells (instead of live cells) for the hybridization of the antisense probes to the RNA molecules [72].

Oligonucleotide-mediated proximity-interactome MAPping (O-MAP) combines RNA-FISH oligonucleotide probes and proximity biotinylating to identify RBPs. Oligonucleotide probes containing a universal “landing pad” sequence are annealed to the RNA of interest in fixed cells. Horseradish Peroxidase (HRP) conjugated to a secondary oligo is then delivered to the universal sequence. Like other PBL enzymes, in the presence of biotin-tyramide and H_2_O_2_, HRP biotinylates molecules surrounding the transcript of interest. This method can identify various parts of the RNA’s interactome like other RNAs through sequencing (O-MAP-Seq), genomic loci (O-MAP-ChIP), and proteins (O-MAP-MS) [73].

**Figure 6:**
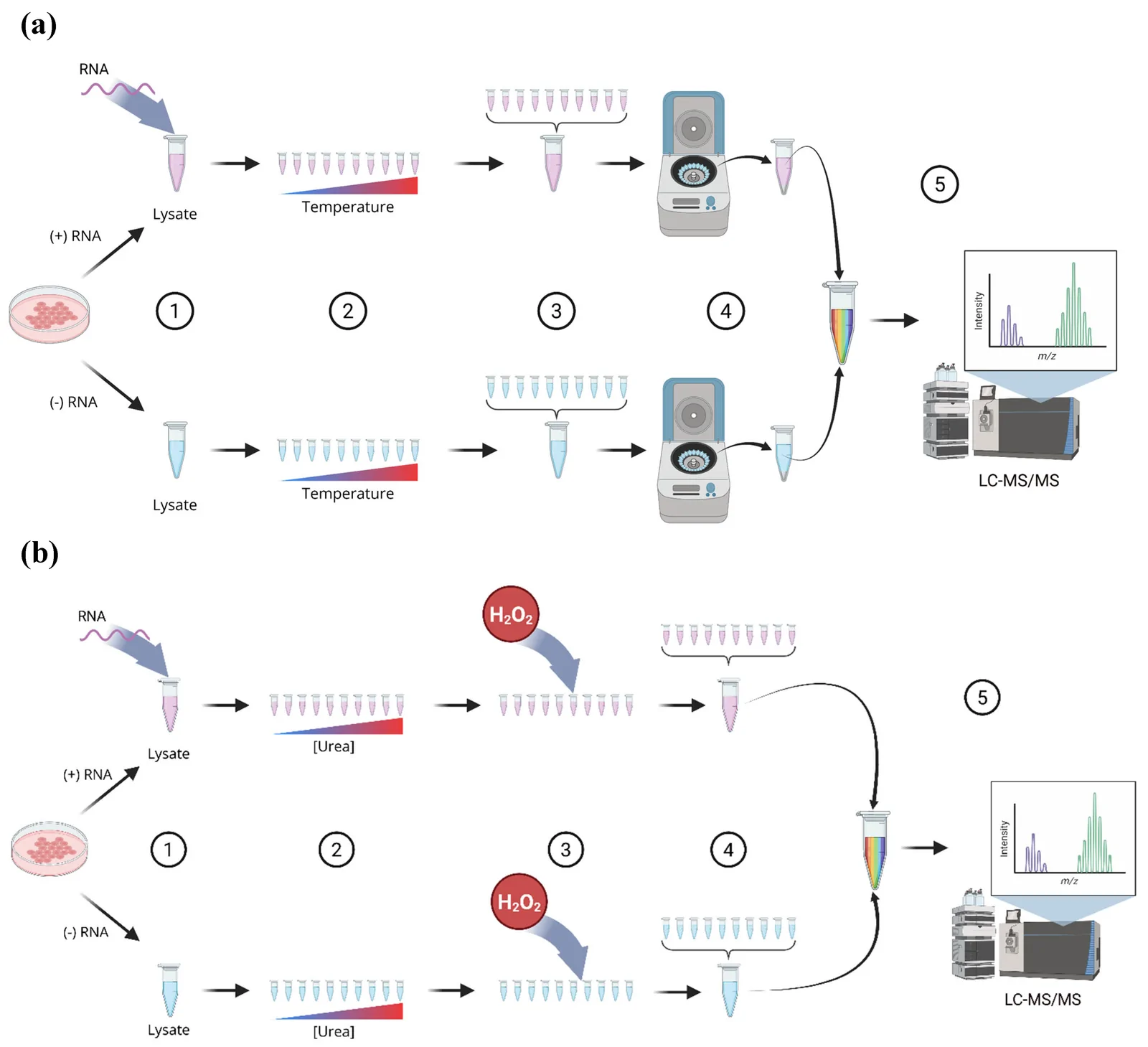
General workflow of proteome-wide stability assays. (**a**) TPP workflow: (1) Cell lysate is split into two pools: one treated with the ligand (+) RNA and one control (−) RNA. (2) Samples are divided into aliquots and heated across a temperature gradient. After equilibration and cooling, (3) the samples are centrifuged to precipitate denatured proteins, and (4–5) peptides are labeled with distinctive isobaric mass tags and pooled into a single analytical sample for mass spectrometry. (**b**) (1) Cell lysate is split into two pools: one treated with the ligand (+) RNA and one control (−) RNA. (2) Aliquots are exposed to increasing concentrations of chemical denaturant to induce unfolding. (3–5) Samples are subjected to methionine oxidation, and after reactions are quenched, peptides are tagged with isobaric labels before pooling. Methionine-containing peptides are enriched using a Pi3 kit prior to MS analysis [74]. Created with BioRender.com (accessed on 21 February 2026).

MS-based techniques have allowed characterization of protein folding and stability in crude biological samples like cell lysates. Examples of these methods involve thermal proteome profiling (TPP), stability of protein from rates of oxidation (SPROX), limited proteolysis (LiP), etc. (Figure 6). Recently, some of these techniques have been adapted to elucidate the protein interactome of an RNA of interest, using unmodified RNA molecules to study RNA–protein interactions of various affinities. The principle behind these proteome-wide stability assays is the change in conformational and thermodynamic stability when an RNA ligand binds a protein, making the protein less susceptible to denaturation or proteolysis. This set of methods subject the lysate with/without the target RNA to denaturant conditions and proceed to analyze them via LC-MS/MS. For example, TPP utilizes a temperature gradient to denature aliquots of lysate proteins in the presence/absence of RNA ligand. The samples are then centrifuged, and the soluble protein fractions from the different aliquots are combined into a single tube to be analyzed via LC/MS-MS-based quantitative bottom-up proteomics. SPROX utilizes a urea concentration gradient as a denaturant. After exposing the aliquots of RNA/RNA-free protein lysate to urea, a methionine oxidation reaction is initiated by the addition of H_2_O_2._ Once the reaction is quenched, the gradient aliquots are combined into a single tube and prepared for LC-MS/MS quantitative bottom-up proteomics. By comparing the readout of the control (−) against the RNA-containing (+) samples, the proteins that are bound to RNA can be determined. Consequently, the identification of proteins that interact with the target RNA relies on shifts in protein stability/oxidation transitions. SPROX monitors protein oxidation rates via a peptide-level readout, whereas TPP relies on thermal profiling to provide a protein-level readout [74]. The identified RBPs can be validated by orthogonal methods such as pull-down and CLIP methods.

### 2.5. In Silico Methods to Identify RBPs

In silico approaches to identify RBPs differ from traditional in vivo and in vitro methods in that they rely on computational data rather than experimental testing. These methods use algorithms that have been trained on experimentally validated data. A variety of methods have been developed following this template as seen in Table 5. An important advantage of in silico approaches is their ability to predict genome-wide RNA-RBP interaction candidates rapidly and cost-effectively. For example, bipartite motif finder (BMF) is the first thermodynamic tool used to identifying bipartite RNA motifs and model the binding affinity of RBPs, and it has demonstrated to detect short and degenerate motifs [4].

One of the recent in silico tools used to detect these interactions is the fRNC (framework of RBP-ncRNA Circuits). This is a systems biology tool designed to identify condition-specific interaction circuits. These predictions are done by integrating transcriptomic, proteomic, and interatomic data. It constructs an RBP–ncRNA network using experimental datasets and can predict RBP-RNA networks in diseases with high confidence [75].

**Table 5:** In silico methods to identify RBPs.

Function	Method	Advantages	Limitations	Citation
Motif Discovery and Scanning	EDCNN	Achieves a high average AUC score; effectively scans whole-genome datasets for structural and sequence motifs.	High computational complexity and time demands.	[76]
	RNAelem	Mitigates false matches via optimized weight models; uses theoretical secondary structure space to discover novel motifs; high Area Under the Receiver Operating Characteristic curve (AUROC).	Susceptible to some false matching; profile context-free grammar (CFG) models do not account for deletions.	[77]
General Interaction Binding and Network Prediction	BMF	Identifies diverse sequence types (degenerate, short, complex, repetitive); models in vivo binding from in vitro data; handles synthetic and real datasets.	Ignores secondary and tertiary structures; requires a core size of 3 for optimal specificity.	[4]
	fRNC	Highly customizable parameters (stringency, network size, scoring); performs well with small networks; elucidates novel mechanistic interactions.	Highly dependent on experimentally derived data; requires a specific network size (~30) for optimal results.	[75]
	GGCRB	Fuses GCN and GAT for enhanced accuracy; integrates sequence and secondary structure features; models long-range and temporal relationships.	Heavily reliant on structural data; misses dynamic/tertiary structures; poor generalizability for rare interactions.	[16]
	MFNN	High prediction accuracy compared to traditional deep kernel models by combining matrix factorization with neural network.	Struggles to accurately predict interactions for circRNAs or RBPs with sparse interaction data.	[78]
	HDRNet	Robustly predicts RBP interactions across diverse cellular conditions and tissues.	Encoding method restricts resolution to larger blocks rather than single nucleotides.	[79]
	scRAPID	Identifies multiple interaction types (RNA-RBP, RBP-RBP) as well as hub RBPs. RBP-RNA interactions are successfully predicted from single-cell transcriptomic data.	Requires massive datasets; gene regulatory network (GRN) performance varies heavily by dataset.	[80]
	RMDNet	Features adaptable feature integration via the improved dung beetle optimization (IDBO) algorithm.	Subject to inherent randomness stemming from population intelligence algorithms.	[81]
	DeepRiPe	Successfully characterizes in vivo RBP targets.	Extracted filters may lack specificity for individual RBPs.	[82]
	Hierarchical DBP/RBP	Capable of predicting novel DBPs and RBPs simultaneously.	When RBPs are included in the dataset, the model’s accuracy in predicting single-stranded DNA-binding proteins (SSBs) drops significantly.	[83]
	PrismNet	Accurately predicts dynamic RNA-binding protein (RBP) interactions by integrating experimental in vivo RNA secondary structure data.	Validation and training are restricted to a limited subset of seven cell lines.	[84]
Alternative Splicing Event Identification	AGML	Uses multi-view similarity matrices to preserve intrinsic data structures and identify candidate RBPs associated with alternative splicing events.	Prone to computational errors when using large projection sizes.	[85]
	RAIMC	Reduces prediction noise using an inductive matrix completion framework and sparse similarity modeling to predict RBPs and alternative splicing event associations.	The integration of multiple kernels can negatively impact specific prediction performance.	[86]
	WDFSMF	Adaptable to other biological entities; weighting system integrates multiple data sources while minimizing background noise to help predict RBP-alternative splicing event associations.	Susceptible to false positives; highly sensitive to specific parameter settings (e.g., k1 value).	[87]
Circular RNA-Specific Interaction and Feature Extraction	MSTCRB	Extracts multi-scale features; integrates a transformer architecture; applicable to both circRNA and linear RNA datasets.	High computational complexity; pipeline dependency.	[88]
	DMSK	Multi-view feature extraction improves recognition accuracy while reducing dimensionality in order to identify interaction sites between circRNAs and RBPs.	Computationally intensive; limited by sparse circRNA data; feature reduction risks losing discriminative information.	[89]
	circTCA	Enhances feature utilization via a cross multi-head attention mechanism to predict circRNA-RBP binding sites	Performance degrades (or produces errors) if an excessive number of attention heads is used.	[90]
	MGFCRSites	Uniquely incorporates chemical molecular structural patterns for highly precise predictions of RBP binding sites on circRNAs.	Susceptible to overfitting due to the reliance on residual blocks.	[91]
	CRIECNN	Utilizes four unique encoding techniques for robust feature extraction for the precise forecasting of circRNA-RBP binding sites.	Training of this model is constrained due to the limited amount of experimentally validated data available for circRNAs.	[29]
	RBPsuite2.0	An accessible web-based interface tool design to predict RBP binding sites across both linear and circular sequences for seven species (human, mouse, zebrafish, fly, worm, yeast, *Arabidopsis*).	Limited to predicting RBPs that already have known binding targets.	[92]
	circRB	Enhances binding site recognition by capturing the directional equivalence of binding orientations on circRNAs.	Provides only marginal AUC improvements (~0.03) over baseline comparative models.	[93]
	SSCRB	Computationally lightweight and resource-efficient for the prediction of interaction sites between circRNA and RBPs.	Relies on outdated programs for base-pairing generation; suffers from positive/negative sample imbalance.	[94]
	CRAFT	Highly user-friendly tool for predicting circRNA function, including interactions with miRNAs and RBP as well as circRNA coding potential; automatically generates HTML pages with interactive tables and figures.	Prone to downstream errors if the underlying CircExtractor tool makes incorrect predictions.	[95]
Pretraining and Representation Training	mRNABERT	Benefits from robust pretraining on one of the largest available mRNA datasets.	Lack of explicit structural modeling and limitations in handling complex genomic features.	[96]
Secondary Structure Generation	CRBPSA	Novel architecture captures complex base-pairing and spatial positional dependencies for accurate secondary structure generation.	Computational intensity of structural calculations; lack of 3D spatial context.	[97]
Interpretability and Feature Analysis	CR-deal	Highly interpretable; tracks integrated gradients to explain feature binding mechanisms.	Sensitive to hyperparameter tuning (e.g., learning rates and batch sizes).	[98]
Ortholog Analysis	PhyloPGM	Reduces false positive rates by incorporating human ortholog sequences.	Restricted exclusively to human species data.	[99]
RNA Localization Prediction	DeepLocRNA	Blend primary sequence information with RBP binding priors in order to predict RNA localization. Capable of cross-species predictions in both mouse and human models.	Optimal accuracy is currently limited strictly to miRNAs and mRNAs.	[100]

Footnote: The methods from 2020 to 2025 publication date in English language were searched using key terms “RBP” (in title/abstract) and “In silico” (in title/abstract) in PUBMED. ((“2020”[Date—Publication]: “2025”[Date—Publication]) AND (English [Language]) AND (RBP [Title/Abstract]) AND (in silico [Title/Abstract])). All abbreviations and their definitions and expanded forms are listed in Supplementary Table S2.

Despite their advantages, existing in silico methods have limitations, including high dimensionality, data sparsity, and low model performance [76]. Recently, motif discovery tools employing deep neural networks have been developed to predict RBP binding sites, by incorporating both sequence and secondary structure information. Deep convolutional neural networks are particularly effective for high-dimensional, sparse data [4,76]. However, as the number of model parameters and network complexity increase, so does the risk of prediction errors, especially since RBPs often bind to low-complexity, untranslated regions of RNA with lower binding affinities [4].

To improve the performance, an evolutionary deep convolutional neural network (EDCNN) was developed to identify protein–RNA interactions by combining evolutionary algorithms and different gradient descent models [76]. Traditional examples of databases include RBPDB and catRAPID, which are used to search for RNA sequences that match RBPs’ binding preferences and to calculate the binding properties between proteins and RNA, respectively [21]. A variety of newer datasets that build from this concept are detailed in Table 6. RNAelem also uses deep neural networks and is designed to predict sequence–structure motifs in the binding regions of RBPs with high accuracy, as demonstrated using simulated data [77].

Tools to predict circRNA-RBP interactions: A graph neural network approach for predicting circRNA–RBP (GGCRB) model integrates nucleotide sequence information with RNA secondary features through graph convolution and attention mechanism to achieve a comprehensive characterization of multiscale features of circular RNAs. Overall, the GGCRB model offers a new alternative for circRNA-RBP prediction, capturing local and global features of circRNAs and achieving an AUC-ROC of 0.9634 [16].

In addition to sequence-based AI prediction methods like RBPSuite and PrismNet, recent years have seen the development of structure-based prediction tools, including AlphaFold3 and RoseTTAFold (NA and All-Atom), as well as biophysics-based tools like PSBHunter, which are comprehensively detailed in the following review [101]. Recently, a new multi-omics database called knockRBP (https://knockrbp.xu-bioinfo.com) was launched. It integrates 1453 datasets across 639 RBPs, 182 cell lines, and 61 diseases, offering a freely accessible resource to facilitate the study of RBP roles in human disease [102].

**Table 6:** Databases for the functional annotation and structural analysis of RBPs and RNA targets.

Database	Description	Link	Citations
**RASP 2.0**	An expanded database of 438 RNA structure probing datasets. Features tools for missing structure score imputation, RBP binding prediction, and RNA secondary/tertiary structure modeling.	http://rasp2.zhanglab.net/	[103]
**QUADRatlas** **v1.2.2401.**	A database specifically designed to include RNA G-quadruplexes (RG4s) and any RBPs that interact with them.	https://rg4db.cibio.unitn.it/	[104]
**RMVar 2.0**	A database that catalogs RNA modifications and annotations for RBP, RNA, and circular RNA interactions. Information on splicing events is also included.	https://rmvar.renlab.cn/	[105]
**EuRBPDB 1.2**	A database including information and functional annotations on eukaryotic RBPs.	http://eurbpdb.gzsys.org.cn/	[106]
**RBPTD** **0.1.0 (2018.9)**	A database that specializes in providing information on cancer-related RBPs.	http://www.rbptd.com/#/	[107]
**RBP-Tar** **2024**	A database that organizes experimental data about RBPs from eCLIP.	https://rbp-tar.biodata.ceitec.cz/	[108]
**OncoSplicing 3.0**	Compiles information on RBPs that play a role in regulation of an individual alternative splicing event.	http://www.oncosplicing.com/	[109]
**POSTAR3**	A platform capable of generating a comprehensive map of RBP binding sites using large scale CLIP-seq datasets and external functional genomic annotations.	http://postar.ncrnalab.org	[110]
**RBP image Database** **2023**	Database mapping 301 RBPs in HepG2 and HeLa cells, featuring immunofluorescence images of RBP-organelle marker pairs alongside systematic localization annotations.	https://rnabiology.ircm.qc.ca/RBPImage/	[111]
**NPInter v5.0**	A ncRNA database featuring an RBP module to display RBP-RNA interactions. Other updates include RNA-DNA interactions and integrated SARS-CoV-2-RNA interactions.	http://bidata.ibp.ac.cn/npinter5/	[112]
**RBP2GO 2.0**	A database that includes information obtained from 53 studies and 105 datasets on RBPs from 13 different species.	https://rbp2gov2.dkfz.de/	[113]
**SliceIt** **2020**	Online database and visualization tool used to design CRISPR/Cas9 screens. It helps to map and plan the editing of protein–RNA interaction sites across the human genome.	http://sliceit.soic.iupui.edu/	[114]
**oRNAment** **2020**	A compilation of putative RBP sites derived from RNAcompete and RNA Bind- n- Seq.	https://rnabiology.ircm.qc.ca/oRNAment	[115]
**EVPsort** **2024**	A specialized database which focuses on ncRNAs found in extracellular vesicles and particles. Information on interactions between ncRNAs and RBPs is included.	https://bioinfo.vanderbilt.edu/evpsort/	[116]

Footnote: The databases from 2020 to 2025 publication date in English language were searched using key terms “RBP” (in title/abstract) and “Database” (in title/abstract) in PUBMED. ((“2020”[Date—Publication]: “2025”[Date—Publication]) AND (English [Language]) AND (RBP [Title/Abstract]) AND (Database [Title/Abstract])). All abbreviations and their definitions and expanded forms are listed in Supplementary Table S2. All the URLs were last accessed on 31 July 2026.

## 3. Clinical and Translational Applications of RNA–Protein Interactions:

RNA–protein interactions play central roles in numerous diseases and the methods to study RBP-RNA interactions have been instrumental in not only identifying the mechanistic details of these interactions but also utilize these interactions as therapeutic targets.

### 3.1. RBP Dysregulation in Human Pathologies

RBPs act as master orchestrators of post-transcriptional gene regulation. Consequently, aberrant expression, mutation, or mis localization of RBPs is linked to a broad spectrum of biological disorders, especially neurodegenerative disorders, cancers, diabetes, and cardiovascular and genetic conditions [12,117].

Neurodegenerative diseases: Pathological aggregation and phase separation of RBPs are hallmarks of neurodegeneration. For example, the RBPs TDP-43 and FUS/TLS are known to play physiological roles in RNA processing, including splicing, transport, and stability, thereby driving the pathogenesis of amyotrophic lateral sclerosis and frontotemporal dementia [118].

Oncology: Dysregulated splicing factors, such as serine/arginine-rich (SR) proteins (e.g., SRSF1, SRSF3, SRSF6), drive oncogenesis by reprogramming alternative splicing. Splicing factors are rarely mutated but often show changes at the expression level. For example, TRA2β (an SR-like protein) is overexpressed in a variety of cancer types, including lung, breast, ovarian, and cervical cancers. On the other hand, heterogeneous nuclear ribonucleoproteins (hnRNPs) act as antagonists of SR proteins to regulate alternative splicing events. However, hnRNPs can also exhibit oncogenic functions (e.g., hnRNPK, hnRNPH1) [119,120].

Diabetes and cardiovascular diseases: Polymorphisms in RBPs are strongly linked to diabetes. For example, the RBP hnRNP F promotes the progression of diabetic kidney disease by regulating angiotensin and transforming growth factor beta 1 [121]. Additionally, hnRNP F mediates the alternative splicing of sarcomeric genes, influencing downstream cardiovascular pathology [122].

Host–Pathogen Interactomes: RBPs also play important roles in the viral replication cycle and in the severity of pathogenesis, as viral RNAs interact with many RBPs, facilitating innate immune evasion, host and tissue tropism, packaging, and egress. Viral translation is strategically enhanced by hijacking host RBPs such as SND1, Staufen1, and Mushashi-1, which mediate transcription initiation of SARS-CoV-2, enhance ribosomal recruitment in enterovirus, and promote Zika virus neurotropism, respectively [123,124].

### 3.2. Therapeutic Targeting of the RBP-RNA Interface

Mapping RBP-RNA interactomes at high resolution has transformed these regulatory interfaces into viable pharmacological targets. Regarding therapeutic strategies, some involve upregulation of RBPs via viral vectors or plasmid-based delivery systems, or knockdown of RBPs using short interfering RNA (siRNA) or antisense oligonucleotides (ASO) [117]. However, siRNA and ASO can also be used to promote the degradation of the target mRNA of RBPs and to modulate RBP binding and RNA splicing, respectively. For example, nusinersen is an antisense drug being used to treat spinal muscular atrophy and can be applied to other neurological diseases [117,125].

Some indirect approaches involve circular RNAs, siRNAs, synthetic peptides, oligonucleotide-based therapies, aptamers, and small molecules [117]. Circular RNAs sequester RBPs, preventing them from binding to endogenous RNA targets and thereby alleviating disease phenotypes caused by pathogenic RBPs [13,117]. Similarly, synthetic peptides, aptamers, and small molecules can also disrupt the interaction between RNA and RBPs by targeting the RBPs [117,126]. Examples of small molecules that bind to RNAs implicated in cancer and neuromuscular and neurodegenerative diseases include compounds at early discovery (e.g., PA-1, cugamycin), phase I/II clinical trials (e.g., zotatifin, branaplam), and FDA-approved (e.g., risdiplam). Risdiplam is the first FDA-approved small molecule targeting SMN2 pre-mRNA exon 7-intron junction that is used to correct splicing defects in spinal muscular atrophy (SMA) [127,128].

RIBOTACs (ribonuclease targeting chimeras) are a new class of small molecules that have been developed for targeted degradation of RNA by recruiting RNase L as the effector and are composed of an RNA-binding module and an RNase-recruiting module that selectively mediates RNA decay [127,129,130]. Compared with oligonucleotide-based therapeutics, RIBOTACs may have superior pharmacokinetic properties. However, RIBOTACs are more challenging to develop than oligonucleotide-based therapeutics [130].

### 3.3. Biomarker Discovery and Low-Input Clinical Profiling

RBPs influence the hallmarks of cancer and multiple other diseases by regulating angiogenesis, tumor-promoting inflammation, immune evasion, genomic instability, among others, and they hold great promise as biomarkers due to their versatile expression in pathological conditions such as RBM38 in melanoma and RBFOX2 in pancreatic cancer [131]. Other examples of RBPs and their roles in disease are shown in Table 7, and known non-coding RNA-RBP interactions in disease are listed in Supplementary Table S1.

Using RBPs as biomarkers in liquid biopsies remains challenging due to their intracellular localization and the difficulty of detecting subtle gene expression changes in clinical samples. Furthermore, RBPs are typically low in abundance in biological fluids [148,149]. Therefore, most biomarker discovery studies focus on upregulated rather than downregulated biomarkers, as the former are easier to validate clinically. In order for an RBP to meet the criteria for a biomarker, it must demonstrate consistent differential expression patterns in both tissue and body fluids under normal versus pathological conditions [150].

To address these detection challenges and map RBP functional roles in low-abundance or limited clinical samples, researchers have increasingly turned to specialized RBP-interaction profiling technologies. Although there have been significant improvements in CLIP methods, they remain labor-intensive and require large sample inputs. Recent innovations such as RT&Tag (~100,000 cells) [151] and ARTR-seq (as few as 20 cells) [152] have facilitated the capture of RBP-interacting transcripts from low-input samples [38]. For example, assay of reverse transcription-based RBP binding site sequencing (ARTR-seq) enables efficient and specific identification of RBP binding sites by relying on in situ reverse transcription of RNAs bound to RBPs and by using antibodies to guide the identification of these sites. It enables the capture of dynamic RNA binding by RBPs over short timescales (e.g., as little as 10 min during stress granule assembly) and is highly efficient for identifying binding sites in cell lines, tissues, and even clinical formaldehyde-fixed samples. Although ARTR-seq avoids material loss by bypassing the antibody-based IP step, a high-quality primary antibody is required to accurately capture RBP-RNA interactions. Another limitation of the method could be the short sequencing length, with an average of 60 bp, and low-abundance RBPs [152,153].

To further resolve cell-type heterogeneity, protein-centric methods coupled with single-cell capture such as STAMP (Surveying Targets by APOBEC-Mediated Profiling) enable the identification of cell-type and RBP-specific RNA–protein interactions across multiple targets within a single, pooled experiment [149]. A related variant, TRIBE-STAMP, allows for simultaneous single-molecule analysis of RBP target pairs within individual cells [154]. Furthermore, MAPIT-seq and INSCRIBE are both protein-centric methods used to profile RBP interactions without requiring genetic manipulation of the organism by using antibody-directed RNA editing to label RNA targets. MAPIT-seq is a new platform that allows direct linking of RBP binding to gene expression outcomes in tissue sections and single cells (~500 cells). Because it adapts well to small tissue samples, it is of particular interest for investigating RBP regulatory mechanisms in clinical samples. Moreover, this scalable protocol supports analysis of multiple RBPs across various samples, making it of interest for large-scale investigation in clinical settings. A single-cell variant of this, scMAPIT-seq, further facilitates the comprehensive interrogation of cell-type-specific RBP functions during development and disease progression [38]. Unlike INSCRIBE technology, MAPIT-seq does not require customized nanobodies for each antibody and enables single-cell resolution and simultaneous co-profiling of the transcriptome and RBP-RNA interactome. However, INSCRIBE has remarkably high sensitivity and enables transcriptome-wide capture of RBP-RNA interactions with low sequencing depth (as low as 20 million reads) or ultra-low input material (~5 mammalian cells). This makes INSCRIBE particularly beneficial for precious clinical specimens and for mapping RBP dynamics in disease contexts [6,38].

To build upon these technologies, future studies should combine multiple omics approaches, incorporating transcriptomic, proteomic and metabolic data, to extract meaningful clinical insights from large-scale datasets [155,156]. Additionally, integrating spatial transcriptomics with H&E staining and protein co-detection will enhance multi-omic profiling, empowering biomedical research to elucidate complex RBP molecular mechanism and drive the discovery of novel therapeutic targets [157,158,159].

## 4. Integrated Workflows and Reliability: Bridging Experimental and In Silico Approaches

A major advancement in this field would be the integration of bioinformatics tools and artificial intelligence with experimental methods. Training computational models in high-quality experimental data enables more precise predictions of binding specificity, RBP-RNA interaction fidelity, and co-binding partners. Computational tools also provide deeper insight into how these interactions function within disease states. Ultimately, the future of RBP discovery lies in a mixed-methods paradigm, in which computational predictions are used to prioritize statistically significant candidate proteins, while high-resolution experimental techniques validate these predictions. For example, deep learning models and RNA–protein interface databases have become widely used in the last couple of years because they have improved approaches for predicting RNA–protein interactions and their binding sites, and they help narrow down the list of candidates [2,77]. Nevertheless, experimental validation remains essential to confirm these in silico findings.

### 4.1. Defining and Evaluating Reliability Between Methods

A primary challenge in decoding RBP dynamics is the discordance observed between computational predictions and experimental readouts, as well as between different experimental platforms.

Experimental Reliability Criteria: Confident identification of bona fide RNA–protein interactions requires validating RBPs discovered via RNA-centric methods using complementary protein-centric approaches. Datasets should be evaluated for positional reproducibility across biological replicates and for signal-to-noise ratios relative to non-specific background controls, such as size-matched input or IgG controls. Critical experimental parameters to account for include antibody specificity, IP efficiency, sequencing depth, and crosslinking efficiency (~5%) [32,44,160,161,162,163]. Furthermore, orthogonal methods such as RT-qPCR following immunoprecipitation, gel shift assays (e.g., newly developed FluoTag-EMSA), or fluorescence in situ hybridization are essential to confirm these results [164,165,166].

Computational Reliability Criteria: Benchmark metrics include the area under the receiver operating characteristic (AUROC), area under the precision-recall curve (AUPRC), and F1 score, all validated against gold-standard experimental datasets such as ENCODE and eCLIP repositories [84,161,167]. It is equally critical to ensure models do not suffer from overfitting and to evaluate their efficacy in distinguishing pathological (e.g., cancer) samples from normal tissues [168].

Cross-Approach Concordance: High reliability is achieved when in silico sequence and structural predictions align with high stringency crosslinking sites in vivo. Discrepancies often stem from competitive RBP binding, RNA tertiary folding, or post-translational modifications, which are contextual nuances that computational tools may fail to capture without contextual and specialized training data [84,169,170]. Consequently, predictions for uncharacterized RBPs remain particularly challenging when relying solely on existing machine learning and docking-based algorithms [169].

### 4.2. Bidirectional Synergy: Predictive Modeling Meets Experimental Mapping

**Figure 7:**
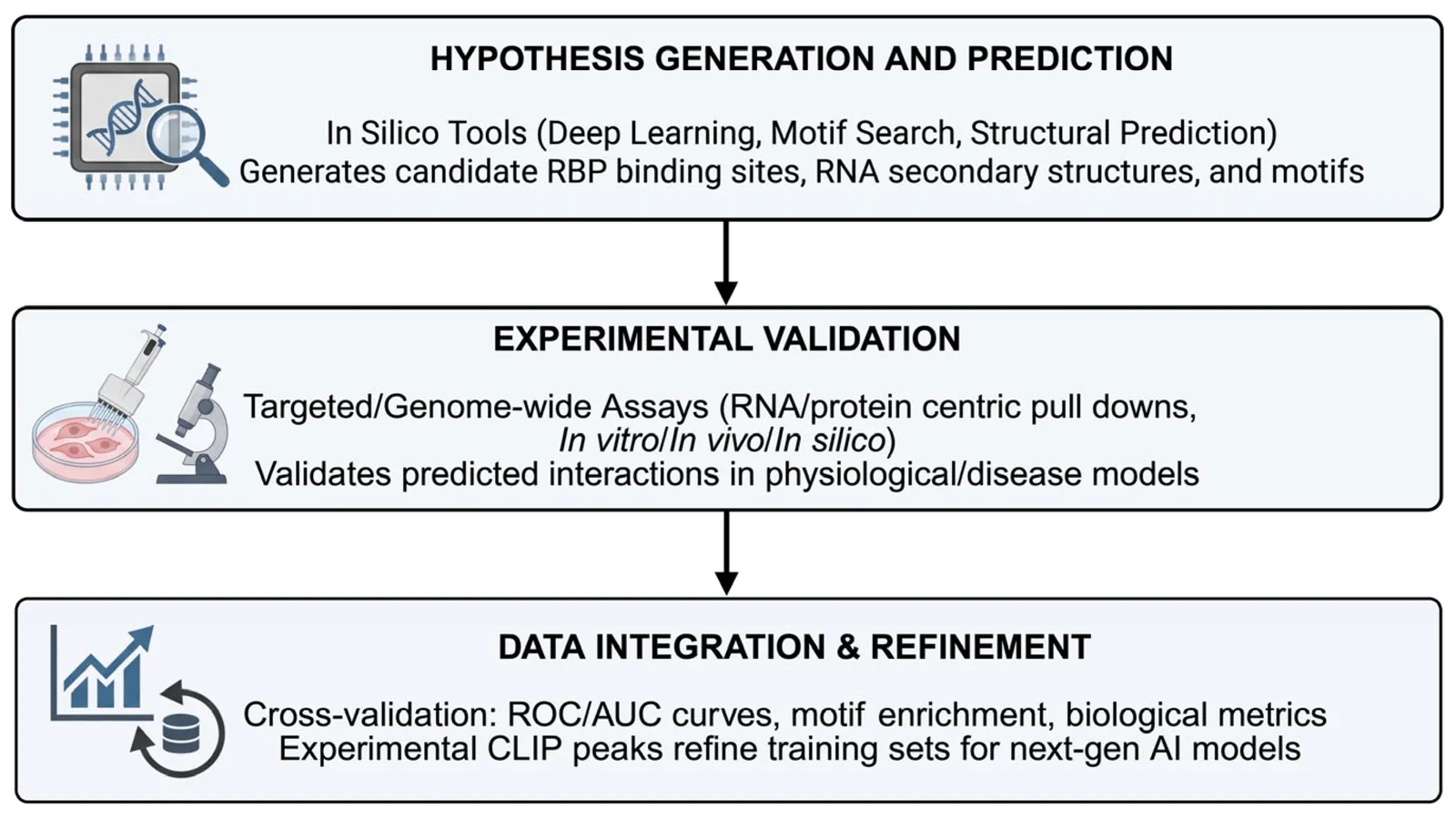
Iterative integrated workflow of interaction between computational and experimental methods.

Rather than operating as isolated strategies, modern experimental and computational techniques form an iterative cycle, as shown in Figure 7.

Computationally Guided Discovery: Machine learning approaches prioritize high-confidence candidate RBP motifs and structural binding pockets [162]. This drastically narrows the scope for experimental mutational screens or targeted pull-down assays [161].

Experimental Benchmark Refinement: Raw sequencing data from high-throughput CLIP-seq and proximity labeling experiments feed back into training datasets. This helps in silico tools account for complex in vivo variables, such as cellular localization, local secondary, and co-factor occupancy [100,161,162].

## 5. Conclusions

**Figure 8:**
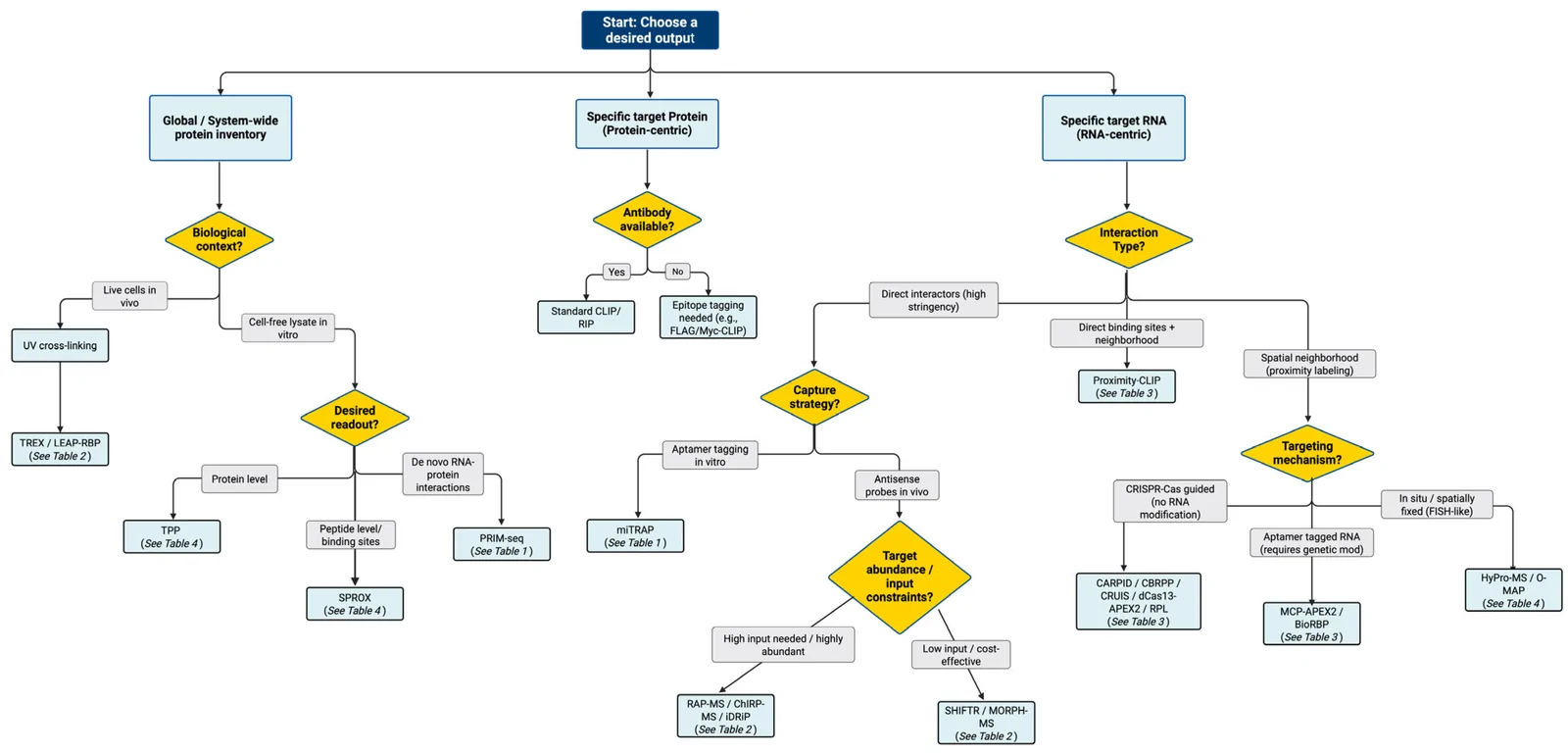
Decision guide for selecting an RNA-binding protein (RBP) identification method. To use this chart, start at the far left by defining your primary desired output: mapping the global interactome, focusing on a specific RNA target, or investigating a specific protein. Follow the branches based on your subsequent experimental constraints, such as whether you require direct binding evidence versus spatial neighborhood mapping, your target’s abundance, and whether your approach is antibody-driven or RNA-centric. Terminal nodes recommend specific methodologies and cross-reference the corresponding comparison tables in this review for detailed advantages, limitations, and cost considerations. Created with BioRender.com (accessed on 21 February 2026).

Global approaches to mapping RNA–protein interactions, such as RIC (RNA interactome capture), OOPS (orthogonal organic phase separation), PTex (phenol-toluol extraction), XRNAX (protein-crosslinked RNA extraction) form the foundation for many of the newer methods described in this review and have been detailed extensively in the previous literature [101,163,171]. Recent advancements in the characterization of RBPs have generated a wide range of experimental and computational platforms. Although these methodologies have expanded our knowledge on RNA-RBP interactions, limitations remain, and there is a particular need for user-friendly approaches to help researchers select appropriate technologies based on their specific needs. Selecting the optimal method for RNA-binding protein (RBP) identification can be challenging given the plethora of new methodologies. To address this, we have provided a practical decision-making guide (Figure 8) tailored to help researchers navigate current experimental and computational options based on experimental design. The first critical step is defining the biological model (immortalized versus primary cells) and the core context of the study. In vitro or ex vivo techniques are highly effective for global, system-level analyses, whereas in vivo and in situ methods are crucial for mapping physiologically relevant interactions in their native environments. Beyond these foundational decisions, the flowchart guides users through practical considerations that ultimately dictate method feasibility: reagent cost, required throughput, experimental turnaround time, and whether the chosen cell model can tolerate the genetic manipulations required by many proximity-labeling techniques.

### 5.1. Current Challenges

Despite rapid technological advances, fundamental challenges remain across the methodological spectrum. A major limitation of current experimental methods is their reliance on immunoprecipitation using antibodies or capture probes. This dependency restricts discovery to known RBPs or those for which specific antibodies or epitope tags are available. Consequently, unconventional RBPs, or RBPs that transiently interact with RNA, may be missed or poorly captured due to antibody non-specificity or binding failure. To unlock the potential of complete RBP discovery and identification, an approach centered on direct, quantitative physical binding, rather than antibody binding, would be required. Furthermore, low crosslinking efficiency, high starting material requirements, and non-specific background binding continue to pose significant hurdles. In terms of computational methods, the major bottlenecks are overreliance on static RNA primary sequences, high false-positive prediction rates in non-coding regions, and limited capacity to model multi-RBP cooperative or competitive complexes dynamically.

### 5.2. Emerging Frontiers

Single-Cell and Spatial RBP Mapping: Combining single-cell sequencing architectures with proximity tagging or targeted CLIP is beginning to unveil cell-type-specific RBP dynamics within complex tissues. Concurrently, integration with spatial transcriptomics will reveal how localized RBP-RNA complexes govern subcellular compartmentalization.

Generative AI and Dynamic Structural Modeling: Advances in sequence-, structure- and biophysics-based predictive tools are transitioning the field from static motif matching toward dynamic 3D structural modeling of coding and non-coding RNA-RBP assemblies.

Integrated Multi-Omics: Future methodologies will routinely link RBP mapping with parallel transcriptomic, proteomic, and metabolomic data, providing a systems-level view of post-transcriptional gene regulation in health and disease.

Overall, individual RBP identification techniques remain limited in their ability to provide a complete picture of the interactome. Optimal experimental designs should cross-validate findings by combining complementary RNA-centric and protein-centric sequencing methods as well as orthogonal validation methods. Ultimately, navigating today’s extensive array of techniques requires balancing the specific biological question against available technical infrastructure and budget constraints.

## Figures and Tables

**Table 2:** In vivo methods—crosslinking-based.

Method	Description	Advantages	Limitations	Cost *	Time (Days)	Throughput **	Citation
RAP-MS	Uses UV crosslinking and long antisense probes (90 nt) to pull down target RNA (e.g., Tau pre-mRNA) and bound RBPs; purified complexes are identified by MS.	Yields high-confidence interactions due to enhanced denaturing/stringent purification; versatile for any RNA.	Is expensive due to individually biotinylated probes, risks losing weaker interactions, needs high input material.	$$$	5–7	Moderate	[48]
iDRiP	Employs crosslinking, biotinylated antisense probes, and hyper-stringent washing to eliminate weak associations and contaminants during RNA affinity purification.	Offers high specificity and low background; yields more specific proteins and fewer mitochondrial contaminants.	Risks losing real interactors with weaker, but functional, binding affinities due to extreme stringency.	$$$	5–7	Moderate	[49]
LEAP-RBP	UV crosslinks proteins to RNA in living cells; isolates complexes using a lithium supplemented solvent that selectively precipitates protein–RNA complexes for MS.	Cost-effective and highly efficient with a quick turnaround time.	Identifies generic RNA Binding proteins, not focused on identifying proteins bound to a specific target RNA.	$	2–3	High	[50]
MORPH-MS	Captures crosslinked target RNA using non-biotinylated tiling antisense oligos pulled down via hybridization to a single biotinylated “Universal oligo” and streptavidin beads.	Serves as a cost-effective adaptation of ChIRP; requires only 1 expensive biotinylated oligo. (the Universal oligo).	Requires optimization of the common binding sequence and antisense oligos tiling array.	$	3–4	Low-moderate	[51]
ChIRP-MS	Crosslinks cells with stronger glutaraldehyde; purifies via biotinylated probes; separates proteins by SDS-PAGE, fractionates the gel, and identifies via MS.	Provides a highly specific method tailored for lncRNAs.	Requires a larger amount of starting input material.	$$$	4–5	Low	[52]
Biochemical Pull-down	Crosslinks cells with formaldehyde, hybridizes with biotinylated DNA probes, extracts with streptavidin beads, reverses crosslinking, and analyzes by MS.	Captures complexes under native/in vivo conditions; maximizes protein yield for MS; offers a fast, robust protocol.	Suffers from a higher amount of non-specific binding and background noise.	$$	3–4	Low	[53]
TREX	UV crosslinks complexes, separating them via TRIZOL/chloroform; anneals tiling DNA oligos, degrades target RNA via RNase H, and extracts free RBPs for quantitative MS.	Unbiased and cost-effective; requires no genetic manipulations and enables endogenous interactome profiling.	Relies on UV crosslinking, risks potential non-specific cleavage, and requires careful oligonucleotide design.	$$	3–4	Moderate	[34]
SHIFTR	UV crosslinks and extracts RNA–protein complexes from interphase using acid guanidinium thiocyanate–phenol–chloroform (AGPC) organic phase separation.; uses DNA probes to pull RNA and RNase H to digest target RNA, shifting proteins to the organic phase for tandem mass tagging (TMT)-based quantitative MS.	Works well with low input material; offers a superior signal-to-noise ratio; detects interactions at specific RNA regions without genetic manipulation.	Remains limited by RNA abundance; may fail to detect transient or indirect interactors.	$$	4–5	Moderate	[54]

Footnote: * Cost scale: Detailed as low ($), moderate ($$), and high ($$$) based on the requirement for specialized reagents, such as individually biotinylated probes versus universal oligos. ** Throughput and time: Estimated turnaround times include cell preparation, crosslinking, purification, and mass spectrometry preparation phases. All abbreviations and their definitions and expanded forms are listed in Supplementary Table S2.

**Table 4:** Other experimental methods to identify RBPs.

Method	Description	Advantages	Limitations	Cost *	Time	Throughput **	Citation
HyPro-MS (in situ)	Hybridization of digoxigenin-labeled antisense probes to target RNA and binding of HyPro, a custom-designed enzyme (fusion of engineered APEX2 and the DIG10.3 domain) that binds digoxigenin and biotinylates proximal proteins and RNA.	No crosslinking required, genetic manipulation, potentially reduced artifacts caused by mislocalization and/or cytotoxicity	Large labeling radius (more false positives), need to control for probe hybridization specificity, probes may compete with RBPs interacting with overlapping target sequences.	$$	2–3 days	Moderate-High	[72]
O-MAP (in situ)	Annealing of RNA-FISH-like probes containing a universal sequence to the target RNA, and subsequent hybridization of a secondary sequence-HRP conjugate that proximity-labels surrounding molecules.	Precise RNA-targeting, no need for genetic manipulation, multiomic application, low cell requirement, uses off-the-self parts.	Challenging for complex FISH targets, formaldehyde fixation may disrupt the subcellular structure and can only identify molecules within the HRP labeling radius.	$$	2 days	High	[73]
TPP (in vitro/ex vivo)	Subjects aliquots of RNA- and vehicle-containing lysates to a temperature gradient to denature the proteins. The respective reactions are combined, and the soluble protein fractions are then analyzed through LC-MS/MS.	No need for RNA modification, analysis of a wide range of interaction affinities, can detect weak/transient interactions, allow for system-level analysis (use of unpurified biological samples).	Less abundant proteins may be missed due to the complexity of the lysate, provides protein-based readout only (compared to SPROX), may miss RBP hits where the RNA ligand denatures before the protein’s thermal melting temperature, may induce RNA unfolding, lower proteomic coverage (compared to SPROX).	$$$	3–4 days	High	[74]
SPROX (in vitro/ex vivo)	Subjects aliquots of RNA- and vehicle-containing lysates to a urea gradient to denature the proteins and a methionine oxidation reaction. The oxidation reaction is quenched, the respective samples are combined, and prepared to be analyzed through LC-MS/MS.	No need for RNA modification, analysis of a wide range of interaction affinities, can detect weak/transient interactions, allow for system-level analysis (use of unpurified biological samples), provides a peptide-based readout (provides domain-specific structural information), high proteomic coverage (compared to TPP), less likely to alter the structure of the RNA molecule.	Less abundant proteins may be missed due to the complexity of the lysate, depends on the protein’s methionine content.	$$$	3–4 days	High	[74]

Footnote: * Cost scale: moderate ($$), and high ($$$) based on the requirement for specialized reagents, such as beads, fusion protein constructs etc. ** Throughput and time: Estimated turnaround times include cell preparation, crosslinking, purification, and mass spectrometry preparation phases. All abbreviations and their definitions and expanded forms are listed in Supplementary Table S2.

**Table 7:** Disease-associated RBPs, pathological mechanisms, and translational horizons.

RBP Name	Primary Biological Function	Associated Disease Context	Mechanism of Dysregulation	Translational/Clinical Horizon	Citation
TDP-43	Splicing regulation, RNA transport, microRNA processing	Amyotrophic lateral sclerosis (ALS); frontotemporal dementia (FTD)	Cytoplasmic aggregation; nuclear depletion; disrupted RNA splicing	ASO-mediated knockdown; small molecule inhibitors; autophagy-activating compounds; vectorized antibody	[118,132,133]
LIN28	Inhibition of the tumor suppressor let-7 miRNA maturation	Stem cell self-renewal and cancer progression (embryonal tumors)	Overexpression and repression of let-7 microRNA biogenesis; regulation of glucose homeostasis and insulin sensitivity	Small molecule inhibitors; PROTAC (Proteolysis Targeting Chimera); molecular glue	[134,135]
HuR	mRNA stabilization, translational enhancement	Multiple solid tumors; cardiovascular disease; neurological pathologies; muscular disorders	Overexpression and cytoplasmic translocation, stabilizing oncogenes	Small molecule inhibitors; PROTAC; shRNA, siRNA, ASO-mediated knockdowns; CRISPR/Cas9-mediated knockout	[131,136,137,138]
SF3B1	Pre-mRNA splicing (U2 snRNP component)	Myelodysplastic neoplasms (MDS), Uveal melanoma	Recurrent point mutations causing aberrant 3’ splice site selection	Spliceosome modulators; synthetic small molecule inhibitors	[139,140]
FUS	Transcriptional regulation, RNA transport, DNA repair	ALS, frototemporal lobar degeneration (FTLD)	Mutations causing aberrant liquid–liquid phase separation and aggregation	ASO targeting FUS transcripts; molecular chaperones	[141,142,143,144]
PTBP1	Repression of alternative splicing, mRNA localization and stability, translational control	Glioma and various cancers including colorectal, liver, lung	Overexpression suppressing neuronal splice patterns, promote the Warburg effect	ASO; small molecule inhibitors; stapled peptides	[145,146,147]

Footnote: All abbreviations and their definitions and expanded forms are listed in Supplementary Table S2.

## Data Availability

No new data were created or analyzed in this study. Data sharing is not applicable to this article.

## Supplementary Materials

The following supporting information can be downloaded at https://www.mdpi.com/article/10.3390/cells15171546/s1, Supplementary Table S1. Examples of known ncRNA-RBP interactions in diseases. Supplementary Table S2: List of Abbreviations and their definitions and expanded forms. Refs. [19,36,37,172,173,174,175,176,177,178,179,180,181,182] are cited in Supplementary Materials.
