# Supplementary material for: Experimental and Computational Approaches to Identify RNA–Protein Interactions
Source: Cells. 2026 Aug 27;15(17):1546. doi: 10.3390/cells15171546 (PMC13564858; doi:10.3390/cells15171546)
Supplement: Supplementary file 1 [file cells-15-01546-s001.zip › cells-4468584-supplementary.pdf]

cells

MDPI

Supplementary Material:

Supplementary Table S1. Examples of known ncRNA-RBP interactions in diseases.

ncRNA	RBP	Relationship	Disease	Citation
LncRNA TPT1-AS1	KHDRBS3	TPT1-AS1 regulates cAMP response element binding protein 1 (CREB1) through binding with RBP KHDRBS3.	Ovarian Cancer (OC)	[1]
CircPBX3	IGF2BP2	CircPBX3 enhances cisplatin resistance in ovarian cancer cells by interacting with RBP IGF2BP2.	Ovarian Cancer (OC)	[2]
miRNA let-7a	DAZL	DAZL upregulates miRNA let-7a in human primordial germ cells.	Essential in germ cell development	[3]
lncRNA Myoparr	hnRNPK	hnRNPK is associated with a decrease in muscle differentiation through repression of myogenin.	Reveals the function of hnRNPK in skeletal muscle cells.	[4]
LINC00152	TRX	TRX is significantly upregulated in HCC, and when it binds to Linc00152, it promotes metastasis.	Hepatocellular Carcinoma (HCC)	[5]
LncRNA HOXC-AS1	IGF2BP2	HOXC-AS1 interacts with IGF2BP2 to stabilizes SIRT1 mRNA expression, which promotes ESCC.	Esophageal squamous cell carcinoma (ESCC)	[6]
LINC00524	TDP43	LINC00524 is a potent amplifier of the growth and metastasis of BC. Interaction with TDP43 strengthens the malignancy of BC cells.	Breast Cancer	[7]
LncRNA WAC antisense RNA 1 (WAC-AS1)	NSUN6	WAC-AS1 is confirmed to have an anti-apoptotic effect on triple-negative breast cancer (TNBC) cells. NSUN6 is an RBP that is related to WAC-AS1 and regulates sensitivity to the drug navitoclax in TNBC cells.	Breast Cancer	[8]

miR-133a-3p	Eef1a1	Eef1a1 promotes the proliferation of myoblasts and prevents their differentiation. This uncontrolled proliferation can often be observed in cancer cells. miR-133a-3p targets Eef1a1 and silences its expression.	Regulation of myogenesis to prevent disease.	[9]
No ncRNA listed, HuR binds to 3′ NCR of RNA in viral and host genomes	HuR (ELAVL1)	HuR increases mRNA stability when bound to its 3′ end.	Crimean-Congo Hemorrhagic Fever virus (CCHFV)	[10]
LncRNA ALMS1-IT1	39 miRNAs and 42 RBPs	ALMS1-IT1 is hypothesized to have a role in the regulation of tumor progression in COAD and may play a role in cell replication.	Colon Adenocarcinoma (COAD)	[11]
lncRNA-RNCR3, Dkc1, and Snora62 and Foxh1	DCP2	DCP2 interacts with RNCR3 to regulate apoptosis by improving RNCR3 stability. Interaction of DCP2 with Snora62, Dkc1, and Foxh1 all promote neuronal apoptosis.	Chronic cerebral ischemia (CCI)	[12]
LncRNA HOTAIR	EZH2 and LSD1	HOTAIR interacts with EZH2 and LSD1	Tumor cell promotion	[13]
miR-16-5p	ELAVL4	ELAVL4 and miR-16-5p exert antagonistic effects on each other regulatory pathways. ELAV4 alleviates AD-associated Aβ aggregation. miR-16-5p targets RBP BCL-2 and promotes Aβ plaque formation and neuronal apoptosis.	Alzheimer's Disease (AD)	[14]

References for Table S1

1. Cao, L.; Wang, Y.; Liu, J.; Bai, X.; Chi, X. Long non-coding RNA TPT1-AS1 inhibits ferroptosis in ovarian cancer by regulating GPX4 via CREB1 regulation. *Am J Reprod Immunol* **2024**, *92*, e13864, doi:10.1111/aji.13864.
2. Fu, L.; Zhang, D.; Yi, N.; Cao, Y.; Wei, Y.; Wang, W.; Li, L. Circular RNA circPBX3 promotes cisplatin resistance of ovarian cancer cells via interacting with IGF2BP2 to stabilize ATP7A mRNA expression. *Hum Cell* **2022**, *35*, 1560-1576, doi:10.1007/s13577-022-00748-8.
3. Yan, A.; Xiong, J.; Zhu, J.; Li, X.; Xu, S.; Feng, X.; Ke, X.; Wang, Z.; Chen, Y.; Wang, H.W.; et al. DAZL regulates proliferation of human primordial germ cells by direct binding to precursor miRNAs and enhances DICER processing activity. *Nucleic Acids Res* **2022**, *50*, 11255-11272, doi:10.1093/nar/gkac856.
4. Hitachi, K.; Kiyofuji, Y.; Nakatani, M.; Tsuchida, K. -Associated and -Independent Multiple Roles of Heterogeneous Nuclear Ribonucleoprotein K during Skeletal Muscle Cell Differentiation. *Int J Mol Sci* **2021**, *23*, doi:10.3390/ijms23010108.
5. Teng, X.; Shang, J.; Du, L.; Huang, W.; Wang, Y.; Liu, M.; Ma, Y.; Wang, M.; Tang, H.; Bai, L. RNA-binding protein Trx regulates alternative splicing and promotes metastasis of HCC via interacting with LINC00152. *J Gastroenterol Hepatol* **2024**, *39*, 2892-2902, doi:10.1111/jgh.16735.
6. Yang, Z.; Wan, J.; Ma, L.; Li, Z.; Yang, R.; Yang, H.; Li, J.; Zhou, F.; Ming, L. Long non-coding RNA HOXC-AS1 exerts its oncogenic effects in esophageal squamous cell carcinoma by interaction with IGF2BP2 to stabilize SIRT1 expression. *J Clin Lab Anal* **2023**, *37*, e24801, doi:10.1002/jcla.24801.
7. Sun, X.; Li, W.; Li, G.; Yang, H.; Jiang, Z.; Shen, L.; Shen, Y.; Liu, Y.; Wang, G. A novel long non-coding RNA LINC00524 facilitates invasion and metastasis through interaction with TDP43 in breast cancer. *J Cell Mol Med* **2024**, *28*, e18275, doi:10.1111/jcmm.18275.
8. Zhou, J.; Zhao, H.; Guo, Y.; Hou, K.; Ding, Q.; Shao, W.; Xu, Q. Comprehensive Analysis of RNA-Binding Protein-Related lncRNA in Breast Invasive Carcinoma. *Front Biosci (Landmark Ed)* **2023**, *28*, 9, doi:10.31083/j.fbl2801009.
9. Liu, W.; Wang, W.; Wang, Z.; Fan, X.; Li, W.; Huang, Y.; Yang, X.; Tang, Z. CRISPR Screen Identifies the RNA-Binding Protein Eef1a1 as a Key Regulator of Myogenesis. *Int J Mol Sci* **2024**, *25*, doi:10.3390/ijms25094816.
10. Ikegawa, M.; Kano, N.; Ori, D.; Fukuta, M.; Hirano, M.; Hewson, R.; Yoshii, K.; Kawai, T.; Kawasaki, T. HuR (ELAVL1) regulates the CCHFV minigenome and HAZV replication by associating with viral genomic RNA. *PLoS Negl Trop Dis* **2024**, *18*, e0012553, doi:10.1371/journal.pntd.0012553.
11. Lin, Y.; Li, Y.; Chen, Y.; Zhang, Z. LncRNA ALMS1-IT1 is a novel prognostic biomarker and correlated with immune infiltrates in colon adenocarcinoma. *Medicine (Baltimore)* **2022**, *101*, e31314, doi:10.1097/MD.0000000000031314.
12. Yang, J.; Liu, X.; Zhao, Y.; Dong, W.; Xue, Y.; Ruan, X.; Wang, P.; Liu, L.; E, T.; Song, J.; et al. Mechanism of Dcp2/RNCR3/Dkc1/Snora62 axis regulating neuronal apoptosis in chronic cerebral ischemia. *Cell Biol Toxicol* **2023**, *39*, 2881-2898, doi:10.1007/s10565-023-09807-8.
13. Yang, D.; Li, C.; Kong, Y.; Pei, Y.; Miao, B.; Dai, G.; Ding, P.; Shi, P.; Wang, Z.; Pei, R. Deciphering the Temporal-Spatial Interactive Heterogeneity of Long Non-Coding RNAs and RNA-Binding Proteins in Living Cells at Single-Cell Resolution. *J Am Chem Soc* **2024**, *146*, 20878-20890, doi:10.1021/jacs.4c05205.
14. Sen, S.; Mukhopadhyay, D. A Holistic Analysis of Alzheimer's Disease-Associated lncRNA Communities Reveals Enhanced lncRNA-miRNA-RBP Regulatory Triad Formation Within Functionally Segregated Clusters. *J Mol Neurosci* **2024**, *74*, 77, doi:10.1007/s12031-024-02244-0.

Supplementary Table S2: List of Abbreviations and their definitions and expanded forms.

Abbreviation	Definition/ Expanded Form
4SU	4-thiouridine
ADAR	Adenosine Deaminase Acting on RNA
AGML	Adaptive Graph-based Multi-Label learning
AGPC	Acid guanidinium thiocyanate–phenol–chloroform
ALS	Amyotrophic Lateral Sclerosis
APOBEC	Apolipoprotein B mRNA editing enzyme, catalytic polypeptide-like
ARTR-seq	Assay of reverse transcription-based RBP binding site sequencing
ASO	Antisense oligonucleotides
AUC-ROC	Area Under the Receiver Operating Characteristic Curve
AUPRC	Area under the precision-recall curve
BMF	Bipartite motif finder
BP	Biotin-phenol
CARPID	CRISPR-Assisted RNA-Protein Interaction Detection
Cas13	CRISPR-associated protein 13
Cas9	CRISPR-associated protein 9
CBRPP	CRISPR-based RNA proximity proteomics
cDNA	Complementary DNA
CFG	context-free grammar
CHART	Capture Hybridization Analysis of RNA Targets
ChIRP	Chromatin Isolation by RNA Purification
circRB	Circular RNA-RBP interaction prediction
circRNAs	Circular RNAs
circTCA	circRNA-RBP interaction prediction based on temporal convolutional network (TCN) and cross multi-head attention mechanism
CLIP	Crosslinking and Immunoprecipitation
CLIP-seq	Crosslinking and Immunoprecipitation followed by sequencing
CR-deal	CircRNA-RBP interaction prediction via Deep Learning
CRAFT	CircRNA Function prediction Tool
CRBPSA	CircRNA-RBP interaction sites identification using sequence structural attention model
CRIECNN	Circular RNA-RBP Interaction predictor using an Ensemble Convolutional Neural Network
CRISPR	Clustered Regularly Interspaced Short Palindromic Repeats
CSD	Cold-shock domain

DBP/RBP	DNA-Binding Protein / RNA-Binding Protein
dCas13	Deficient Cas13
DeepLocRNA	Deep Learning for Subcellular Localization of RNA
DeepRiPe	Deep RBP binding preference
DMSK	Multi-view deep learning, subspace learning and multi-view classifier for the identification of circRNA-RBP interaction sites
dsRBD	Double-stranded RNA-binding domain
dsRNA	Double-stranded RNA
eCLIP	Enhanced Crosslinking and Immunoprecipitation
EDCNN	Evolutionary Deep Convolutional Neural Network
EuRBPDB 1.2	Eukaryotic RNA Binding Protein Database
EVPsort	Extracellular Vesicles and Particles sorting database
FDA	Food and Drug Administration
fRNC	Framework of RBP-ncRNA Circuits
FTD	Frontotemporal dementia
FTLD	Frontotemporal lobar degeneration
FUS	Fused in Sarcoma
GAT	Graph Attention Network
GCN	Graph Convolutional Network
GGCRB	Graph neural network approach for predicting circRNA-RBP interactions
GRN	Gene Regulatory Network
gRNA	Guide RNA
H&E	Hematoxylin and Eosin
HDRNet	High-throughput Dynamic Cellular RNA-binding Event Identification using Deep Neural Network
HeLa	Henrietta Lacks (immortal cell line)
HepG2	Human hepatocellular carcinoma cell line G2
HITS-CLIP	High-Throughput Sequencing of RNA isolated by Crosslinking Immunoprecipitation
HRP	Horseradish Peroxidase
HuR	Human antigen R (also known as ELAVL1)
HyPro	Hybridization-proximity labeling
iCLAP	Individual-nucleotide resolution Crosslinking and Affinity Purification
iCLIP	Individual-nucleotide resolution CrossLinking and Immunoprecipitation
IDBO	Improved dung beetle optimization
IDR	Intrinsically disordered regions

iDRiP	Identification of Direct RNA-interacting Proteins
INSCRIBE	Integrating Single-Cell RNA-Binding Evaluation
KH	K homology domains
LC-MS/MS	Liquid Chromatography with tandem Mass Spectrometry
LEAP-RBP	Liquid Emulsion–Assisted Purification of RNA-Bound Protein
LIN28	RNA binding protein Lin28
LiP	Limited proteolysis
lncRNAs	Long non-coding RNAs
MAPIT-seq	Modification added to RBP interacting transcript-sequencing
MCP	MS2 coat protein
MDS	Myelodysplastic Syndrome
MFNN	Matrix Factorization Neural Network
MGFCRSites	Molecular graph features and residual blocks to predict interaction sites between circRNA and RBP
miRNAs	MicroRNAs
MORPH-MS	Multiple Oligo-assisted RNA Pulldown via Hybridization followed by Mass Spectrometry
mRNA	Messenger RNA
mRNABERT	Messenger RNA Bidirectional Encoder Representations from Transformers
MS	Mass spectrometry
MSTCRB	Multi-Scale Transformer for CircRNA-RBP binding prediction
NCBI	National Center for Biotechnology Information
ncRNA	Non-coding RNA
NPInter v5.0	Noncoding RNA and Protein Interactions database
O-MAP	Oligonucleotide-mediated proximity-interactome mapping
OncoSplicing 3.0	Database for identifying RBPs regulating alternative splicing events in cancers
OOPS	Orthogonal organic phase separation
oRNAment	o RNA motifs enrichment in transcriptomes
PAR-CLIP	Photoactivatable Ribonucleoside-enhanced Crosslinking and Immunoprecipitation
PHD	Pumilio homology domain
PhyloPGM	Phylogenetic Probabilistic Graphical Model
PL	Proximity labeling
POSTAR3	POST-trAnscriptional Regulation database
PRIM-seq	Protein-RNA Interaction Mapping by sequencing
PrismNet	Web server for predicting protein–RNA interaction using in vivo RNA structural information

PROTAC	Proteolysis Targeting Chimera
PTBP1	Polypyrimidine Tract-Binding Protein 1
PTex	Phenol-toluol extraction
QC	Quality Control
qRT-PCR	Quantitative Reverse Transcription Polymerase Chain Reaction
QUADRatlas	RG4 and RG4-binding proteins (RG4BPs) database
RAIMC	Prediction of RNA-binding proteins and alternative splicing associations using Inductive Matrix Completion
RAP-MS	RNA Antisense Purification followed by Mass Spectrometry
RaPID	RNA-protein Interaction Detection
RASP 2.0	RNA Atlas of Structure Probing
RBD	RNA-binding domain
RBP	RNA-Binding Protein
RBP image Database	RNA Binding Protein image Database
RBP-Tar	RNA-Binding Protein Target database
RBP2GO 2.0	RNA Binding Protein to Gene Ontology database
RBPs	RNA-binding proteins
RBPsuite2.0	RNA-Binding Protein Suite version 2.0
RBPTD	RNA Binding Protein in Tumors Database *(officially described as a database of cancer-related RNA-binding proteins)*
RG4	RNA G-quadruplex
RGG	Arginine-glycine-glycine
RIBOTACs	Ribonuclease targeting chimeras
RIC	RNA interactome capture
RIP-seq	RNA Immunoprecipitation followed by sequencing
RMDNet	RNA-aware Multi-branch integration network for RNA-protein binding site prediction
RMVar 2.0	Database of functional variants involved in RNA modifications
RNA-FISH	RNA Fluorescence In Situ Hybridization
RNAelem	Predicts sequence-structure motifs in RBP-binding regions
RNP	Ribonucleoprotein
SARS-CoV-2	Severe Acute Respiratory Syndrome Coronavirus 2
scRAPID	Single-cell RNA-Protein Interaction Detection
SF3B1	Splicing Factor 3B subunit 1
SHIFTR	Selective RNase H-mediated interactome framing for target RNA regions
shRNA	Short Hairpin RNA
siRNA	Small Interfering RNA

siRNAs	Small interfering RNAs
SliceIt	Database of in silico sgRNA library to edit protein-RNA interaction sites in the human genome
SMA	Spinal muscular atrophy
snRNAs	Small nuclear RNAs
snRNP	Small Nuclear Ribonucleoprotein
SPROX	Stability of protein from rates of oxidation
SSBs	Single-stranded DNA-binding proteins
SSCRB	Sequence and Structure-based model for CircRNA-RBP binding
STAMP	Surveying Targets by APOBEC-Mediated Profiling
TDP-43	TAR DNA-Binding Protein 43
TMT	Tandem mass tagging
TPP	Thermal proteome profiling
TREX	Targeted RNase H-mediated extraction of crosslinked RBP
TRIBE	Targets of RNA-binding proteins Identified By Editing
uvCLAP	Ultraviolet Crosslinking and Affinity Purification
WDFSMF	Weighted Data Fusion with Sparse Matrix tri-Factorization
XRNAX	Protein-crosslinked RNA extraction
ZnF	Zinc fingers
